# Genome-Wide Identification of the HD-ZIP III Subfamily in Upland Cotton Reveals the Involvement of GhHB8-5D in the Biosynthesis of Secondary Wall in Fiber and Drought Resistance

**DOI:** 10.3389/fpls.2021.806195

**Published:** 2022-01-27

**Authors:** Jie Zhang, Yanan Gao, Mengru Feng, Yuke Cui, Shuaijie Li, Le Liu, Ye Wang, Wenliang Xu, Fuguang Li

**Affiliations:** ^1^State Key Laboratory of Cotton Biology, Institute of Cotton Research, Chinese Academy of Agricultural Sciences, Anyang, China; ^2^Zhengzhou Research Base, State Key Laboratory of Cotton Biology, School of Agricultural Sciences, Zhengzhou University, Zhengzhou, China; ^3^Hubei Key Laboratory of Genetic Regulation and Integrative Biology, School of Life Sciences, Central China Normal University, Wuhan, China

**Keywords:** cotton (*Gossypium hirsutum*), HD-ZIP III, ectopic expression, secondary wall, drought resistance

## Abstract

A subfamily of transcription factors known as HD-ZIP III plays distinct roles in the secondary cell wall biosynthesis, which could be attributed to the quality of cotton fiber and adaptation to drought stress. In this study, 18 *HD-ZIP III* genes were identified as genome wide from the upland cotton (*Gossypium hirsutum*). These genes are distributed on 14 different chromosomes, and all of them have undergone segmental duplications. Numerous *cis*-elements were identified in the promoter regions, which are related to phytohormone responses and abiotic stresses. Expression profiling of these genes by quantitative real-time (qRT)-PCR illustrated their differential spatial expression, with preferential expression in cotton fiber. Among these genes, GhHB8-5D was predicted to encode a protein that is targeted to the cell nucleus and having self-activation ability. In addition, the ectopic expression of *GhHB8-5D* or its synonymous mutant *GhHB8-5Dm* in Arabidopsis resulted in stunted plant growth, curly leaves, and twisted inflorescence stems. Microscopy examination revealed that the morphology of vascular bundles and deposition of secondary wall had substantially altered in stems, which is concomitant with the significant alteration in the transcription levels of secondary wall-related genes in these transgenic Arabidopsis. Further, ectopic expression of *GhHB8-5D* or *GhHB8-5Dm* in Arabidopsis also led to significant increase in green seedling rate and reduction in root length relative to wild type when the plants were grown under mimicked drought stress conditions. Taken together, our results may shed new light on the functional roles of GhHB8-5D that is attributable for secondary cell wall thickening in response to drought stress. Such a finding may facilitate a novel strategy for improving plant adaptations to environmental changes *via* regulating the biosynthesis of secondary cell wall.

## Introduction

As the most important crop producing natural fibers, upland cotton (*Gossypium hirsutum*) is broadly cultivated in the temperate regions in the world and the quality of fiber is of paramount economic significance (Paterson et al., 2012). Fiber development undergoes four distinctive but overlapping stages: initiation, elongation (primary cell wall biosynthesis), secondary cell wall (SCW) thickening (cellulose biosynthesis), and maturation (Haigler et al., 2012). The formation of SCW in fiber cells has been recognized as one of the most crucial steps that directly affect cotton fiber quality (Li et al., 2015).

In mature cotton fiber, SCW is mainly composed of cellulose that is a polysaccharide synthesized by cellulose synthase (CESA) complex (McFarlane et al., 2014; Hernandez-Gomez et al., 2015). Transcriptional regulation network controlling SCW biosynthesis has been extensively elucidated in the model plant Arabidopsis (*Arabidopsis thaliana*), in which numerous transcription factors (TFs) such as NAC acting as master switch coordinate the expression of *CESA*s by coupling with MYB and others (Taylor-Teeples et al., 2015; Zhong and Ye, 2015). However, in cotton, only a limited number of TFs that regulate SCW synthesis have been identified, while their functionality and intricate relationship remain poorly understood (Zhang et al., 2018a; Huang et al., 2019, 2021; Cao et al., 2020). This is despite the recent premise of a model of a four-layered transcriptional regulatory network consisting of GhTCP4, GhMYB7, GhFSN1, and GhMYB46_D13 regulating fiber SCW *GhCesA* genes (Huang et al., 2021). Such a network model may still fall short in sophistication, as many other TFs that function in concert in SCW formation, such as HD-ZIP III TFs, remain unexplored (Robischon et al., 2011; Du et al., 2015).

HD-ZIP III subfamily contains five members in the *A. thaliana* genome, including REVOLUTA/INTERFASCICULAR FIBERLESS1, ATHB8, PHAVOLUTA/ATHB9, PHABULOSA/ATHB14, and CORONA/ATHB15, all of which harbor a leucine zipper motif (LZ) downstream of the homeodomain (HD) (Schena and Davis, 1992). The HD is responsible for the specific binding to target DNA, whereas LZ acts as a dimerization motif (Ariel et al., 2007). The conserved amino acids then form a START (steroidogenic acute regulatory protein-related lipid transfer) domain and an adjacent conserved region known as SAD (START-adjacent domain). *HD-ZIP III* genes are posttranscriptionally regulated by miR165/166 that targets the START domain of the subfamily (McConnell et al., 2001; Kidner and Martienssen, 2004; Kim et al., 2005). Additionally, all members of this subfamily have a conserved domain known as MEKHLA in their C-termini, which shares significant similarity with the PAS domain and is involved in light, oxygen, and redox potential sensing (Mukherjee and Burglin, 2006).

Previous studies showed that all the members in the HD-ZIP III subfamily were required for xylem cells differentiation and secondary wall biosynthesis. For example, *REV* is a positive regulator of secondary wall deposition in interfascicular fibers, and its defective mutant *rev* lacked normal interfascicular fibers in stems (Zhong et al., 1997; Zhong and Ye, 1999). *REV* is also negatively regulated by KNAT7 and BLH6, the expression of which promoted SCW deposition in the *knat7/blh6* double knockout mutant (Liu et al., 2014). *ATHB8*, a gene positively regulated by auxin (Baima et al., 1995), was considered as an early marker of the procambial and cambial cells during vascular development. Ectopic expression of *ATHB8* in Arabidopsis increased the production of xylem tissues (Baima et al., 2001). Similarly, a *Populus trichocarpa* HD-ZIP III gene, *PtrHB7*, was preferentially expressed in the cambial zone; on the other hand, *PtrHB7*-suppressed plants displayed significant changes in vascular tissues with a reduction in xylem but increase in the phloem (Zhu et al., 2013). In the phylogenetic analysis of *A. thaliana* genes, ATHB9, ATHB14, and REV comprised a clade and exhibited similar expression patterns in the vasculature, with ATHB9 and ATHB14 as a sister pair (McConnell et al., 2001; Emery et al., 2003). Mutations in the *ATHB9* and *ATHB14* genes enhanced the vascular defects of the *rev* mutant (Prigge et al., 2005). The coordinated expression of *REV*, *ATHB9*, and *ATHB14* are necessary for xylem cell specification and secondary wall biosynthesis (Carlsbecker et al., 2010). The overexpression of *OsHB4*, a member of the rice HD-ZIP III subfamily, resulted in leaf rolling and altered stem xylem in rice, and the polysaccharide synthesis-related genes could be regulated by miR166-OsHB4 as revealed by transcriptomic analysis (Zhang et al., 2018b). Therefore, *OsHB4* may contribute to cell wall formation and vascular development in rice. In Arabidopsis, overexpression of a miRNA-resistant *ATHB15* resulted in moderate dwarfing, upcurling leaves, and a drastic reduction in xylem and lignified interfascicular tissues (Kim et al., 2005). Transgenic *Populus* expressing a synthetic miRNA targeting *ATHB15* led to abnormal lignification in cells of the pith, while the overexpression of a miRNA-resistant *ATHB15* caused delayed lignification of xylem and phloem fibers during secondary growth (Du et al., 2011), hence ATHB15 was believed to be involved in secondary wall transcriptional pathway by regulating wall-related TFs and synthetic genes (Du et al., 2015).

The plant root xylem is a specialized tissue that distributes water to the shoot. In Arabidopsis, water deficiency enhanced the levels of miR165, which in turn negatively affected *HD-ZIP III* expression and impacted on xylem development and hydraulic conductivity of root (Ramachandran et al., 2018). On the other hand, the overexpression of a miR166-resistant form of *OsHB4* contributed to cell wall formation, vascular development, and drought resistance in rice (Zhang et al., 2018b), which was well in line with the overexpression of *OsHOX32* in rice that displayed narrow rolled leaves, reduced stomatal conductance, and transpiration rate, leading to the improvement in water use efficiency (Li et al., 2016). In cotton, deep sequencing of salt- and drought-treated small RNA libraries, led to the identification of *ghr-miR166a-j* that was downregulated in both drought and salinity treatments, and its target gene *ghr-HD-ZIP III*s was significantly upregulated as a result (Xie et al., 2015).

In this study, we performed a comprehensive genome-wide analysis of *HD-ZIP III* genes in cotton and presented characteristics of this subfamily. In addition, we further investigated the function of GhHB8-5D in Arabidopsis, overexpression of which altered the morphology of vasculature, secondary wall deposition, and drought tolerance. This study may not only provide new insight on the functions of GhHB8-5D but also facilitate additional useful tools for genetic improvements of cotton fiber quality and drought resistance.

## Materials and Methods

### Identification of *HD-ZIP III* Genes in Upland Cotton

Five protein sequences of Arabidopsis HD-ZIP III members were obtained from TAIR^1^ as queries to search cotton (*Gossypium hirsutum* L. acc. TM-1) genome database^2^. The conserved HD (PF00046), START domain (PF01852), and MEKHLA domain (PF08670) of HD-ZIP III subfamily were found by using Pfam^3^, HMMER^4^, and the Batch Web CD-Search^5^. The molecular features and subcellular localization of the cotton HD-ZIP III proteins were predicted using the ProtParam tool^6^ and Plant-mPLoc^7^, respectively.

### Gene Structure and Conserved Motif Analysis

The genomic and coding sequences of *HD-ZIP III* genes were compared by Gene Structure Display Server^8^ to investigate the distribution of exon/intron. A total number of 18 HD-ZIP III protein sequences were used to predict the conserved motifs by using the MEME online program^9^, which were further validated by the Batch Web CD-Search, Pfam, and Batch SMART^10^.

### Chromosomal Location and Collinearity Analysis

Chromosomal location information of HD-ZIP III genes was extracted from the cotton genome database. All the members of the HD-ZIP III subfamily were mapped to their respective locus of chromosomes and visualized using TBtools (Chen et al., 2020). For collinearity analysis, the protein sequences of 18 *HD-ZIP III* genes were served as queries for BLASTP to search the cotton genome database. Duplicated sequences of *HD-ZIP III* genes were identified as previously described. The alignment covers > 80% of the longer gene and the aligned region has an identity > 80% at the nucleotide level (Wang et al., 2020; Zhang et al., 2020). MCScanX software was employed to estimate the collinear pairs and gene duplication type and the result was visualized by using Circos software (Krzywinski et al., 2009; Wang et al., 2012).

### *Cis*-Element Distribution in Promoter Sequences of *HD-ZIP III* Genes

A fragment of 2 kb upstream region of the transcriptional start site of each *HD-ZIP III* gene was retrieved as the promoter sequence and analyzed using the PlantCARE^11^ for *cis*-element prediction.

### Phylogenetic Analysis

The putative protein sequences of HD-ZIP III derived from *A. thaliana*, *Gossypium arboretum*, *G. hirsutum*, *G. raimondii*, *Oryza Sativa*, *Populus trichocarpa*, and *Zinnia elegans* genes were aligned using ClustalX and a phylogenetic tree was constructed by using MEGA7 and the maximum likelihood method with 1000 bootstrap replications (Larkin et al., 2007; Kumar et al., 2016).

### Plant Materials and Growth Conditions

*Gossypium hirsutum* cv. ZM24 plants were grown to maturation in a controlled growth chamber under 30°C with a 16 h photoperiod. The seeds of *A. thaliana* ecotype Col-0 were surface-sterilized and sown on half strength Murashige and Skoog medium supplemented with 2% sucrose. After a vernalization period at 4°C for 48 h, the plates containing Arabidopsis seeds were transferred to a plant growth incubator for a further 7 days before being transplanted to soil in a greenhouse at 22°C with 16 h photoperiod.

### Vector Construction and Transformation

The entire coding region of *GhHB8-5D* was PCR amplified and subcloned into pBI121 under the transcriptional control of the CaMV 35S promoter. Mutations at the miR166a target site were introduced by fusion PCR amplification to construct the *GhHB8-5Dm* overexpression vector. Two overlapping primers (Supplementary Table 5) that mismatched the miRNA binding sites without changing the protein coding sequence were used to generate mutations in the *GhHB8-5D* cDNA sequence. The mutated cDNA was then inserted into pBI121 behind the CaMV 35S promoter. The vectors were transferred into *Agrobacterium tumefaciens* and introduced in *A. thaliana Col-0* by floral dip method (Zhang et al., 2006). Transgenic Arabidopsis seedlings were selected by germinating seeds on kanamycin-supplemented Murashige and Skoog (MS) agar plates. Two independent lines with a single transgene were bred to homozygosity and used for further analysis.

### Subcellular Localization and Transcription Activation Analysis

The coding sequence of *GhHB8-5D* was cloned to a pBI-eGFP vector that was then introduced into *A. tumefaciens* for transient expression in the leaf tissues of *Nicotiana benthamiana* as previously described (Sparkes et al., 2006). Green fluorescent protein (GFP) in agro-infiltrated *N. benthamiana* leaf cells was detected under an SP5 Meta confocal laser microscope (Leica Microsystems, Mannheim, Germany). To investigate the transcriptional activity of GhHB8-5D, both the full-length and truncated coding sequences of *GhHB8-5D* were inserted into the pGBKT7 vector, which were then introduced into baker’s yeast *Saccharomyces cerevisiae* strain AH109 using the high-efficiency lithium acetate transformation procedure. Yeast transformants were streaked on a selective medium lacking tryptophan to assay transcriptional activity. the β-galactosidase activity was also assayed by colony-lift filter assay using 5-bromo-4-chloro-3-indolyl β-D-galactopyranoside (X-gal) as substrate.

### Stem Sections and Microscopic Analysis

Freehand slicing and paraffin-embedded section of Arabidopsis stems and secondary wall staining were performed as previously described (Huang et al., 2019).

### RNA Isolation and qRT-PCR

Cotton RNA was extracted from different cotton tissues, and *Arabidopsis* RNA was isolated from 6-week-old stems using RNAprep Pure Plant Plus Kit (TIANGEN, Beijing, China) according to the manufacturer’s instructions. Expression profiles of cotton and Arabidopsis genes were analyzed by qRT-PCR using *GhUBI1* (EU604080) and *AtActin2* (AT3G18780.1) as reference genes according to a previously described method (Xu et al., 2013). All primers were listed in Supplementary Table 5.

### Assay of Green Seedling Rate and Primary Root Elongation

Thirty surface-sterilized seeds from wild type or each transgenic Arabidopsis line were placed for germination on the MS medium supplemented with or without different concentrations of mannitol and placed at 4°C for 2 days, prior to being moved to a growth room at 22°C and 16 h photoperiod. The number of seedlings with green cotyledons was counted after 2 days. Each experiment was repeated three times.

For assaying primary root length, the seeds of wild type or transgenic Arabidopsis lines were germinated and maintained on MS medium supplemented with different concentrations of mannitol for 7 days. The length of primary roots of seedlings was measured and compared. All experiments were repeated at least three times.

## Results

### Identification of HD-ZIP III Subfamily Members in Upland Cotton

In order to identify *HD-ZIP III* genes in cotton, five Arabidopsis HD-ZIP III protein sequences were used as queries to blast search the cotton genome database. The putative HD-ZIP III protein sequences were analyzed by Pfam, HMMER, and the Batch Web CD-Search to find conserved domains. As a result, 18 genes were identified as the members of HD-ZIP III subfamily in cotton. Every HD-ZIP III protein encompasses three conserved domains, including the HOMEOBOX domain, START domain, and MEKHLA domain (Supplementary Table 1). The 18 cotton *HD-ZIP III* genes were designated according to their chromosomal positions. The protein length and molecular weights are about 840 amino acids and 92 KDa, respectively, in all these HD-ZIP III proteins except GhHB8-5A, these two features of which are 776 amino acids and 85.675 KDa, respectively. The isoelectric point (pI) of HD-ZIP IIIs varies from 5.74 to 6.19. Bioinformatics analysis predicted that all cotton HD-ZIP III proteins may be located in the nucleus (Supplementary Table 2).

### Gene Structure and Conserved Motifs of Cotton *HD-ZIP III* Genes

**FIGURE 1:**
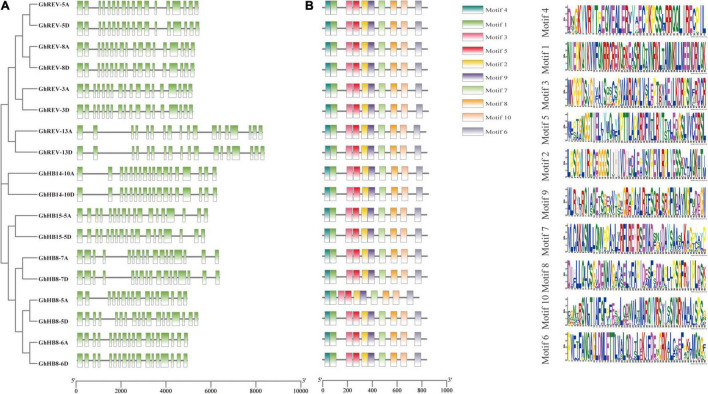
Gene structure and conserved motifs of HD-ZIP III subfamily in cotton (*Gossypium hirsutum*). **(A)** The exon/intron distribution of *HD-ZIP III* genes. Green boxes and black lines represent exons and introns, respectively. **(B)** Conserved motifs of each HD-ZIP III protein. Each conserved motif is indicated by different color boxes. The length of each gene and protein can be estimated using the scale at the bottom.

To get a further understanding of the cotton HD-ZIP III subfamily, gene structure and conserved motifs of each *HD-ZIP III* member were investigated. Most of the *HD-ZIP III* genes possess 17 introns, while *GhHB8-5A*, *GhHB8-7A*, and *GhHB8-7D* have 15, 16, and 16 introns, respectively (Figure 1A). Such a distribution pattern of exon/intron is consistent with that of *HD-ZIP III* genes in rice (Agalou et al., 2008). As shown in Figure 1B, there are ten different motifs in each HD-ZIP III based on the sequence conservation as predicted by using MEME. Among them, motifs 1 and 4 are the core sequences of the HD and LZ domains, respectively. Motif 2, 3, 5, and 9 compose the START domain. The SAD domain is constitutive of motif 7 and 8. Motif 6 and 10 belong to the MEKHLA domain. The fact that all cotton HD-ZIP IIIs share the same batch of motifs is suggestive of functional versatility and potential redundancy in this subfamily.

### *Cis*-Element Analysis in Promoter Sequences of Cotton *HD-ZIP IIIs*

**FIGURE 2:**
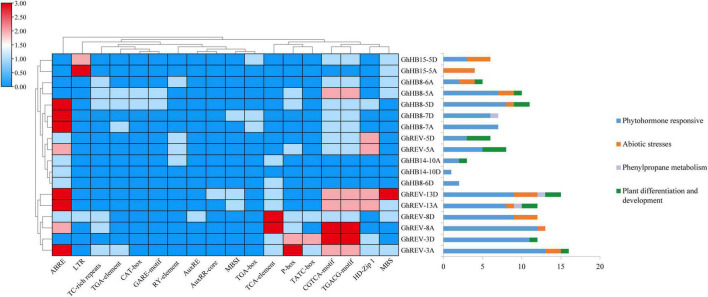
Statistics of *cis*-elements in the promoter region of cotton (*Gossypium hirsutum*) *HD-ZIP III* genes. The left part shows statistics on the number of specific *cis*-elements contained in each *HD-ZIP III* gene promoter region. The right part exhibits statistics on the function of *cis*-elements possessed in each *HD-ZIP III* gene promoter region.

The promoter regions of cotton *HD-ZIP III* genes were analyzed to study their biological function in depth. As shown in Figure 2, in the 2 kb sequences upstream of the start codon, many *cis*-elements were identified, including ABRE, TGA-element, GARE-motif, AuxRE, AuxRR-core, TGA-box, TCA-element, P-box, TATC-box, CGTCA-motif, and TGACG-motif, which were reported to respond to the induction of various phytohormones, while LTR (cis-acting element involved in low-temperature responsiveness), TC-rich repeats (cis-acting element involved in defense and stress responsiveness), and MBS (MYB binding site involved in drought inducibility) were involved in abiotic stresses responsiveness. From the distribution of these *cis*-elements in each *HD-ZIP III* promoter, it is conceivable that almost all cotton *HD-ZIP III*s could be involved in phytohormone responsiveness, such as auxin, gibberellin, and abscisic acid, except *GhHB15-5A*. More than half of this subfamily member could be responsive to drought and low-temperature stresses.

### Chromosomal Location, Gene Duplication, and Phylogenetic Analysis

**FIGURE 3:**
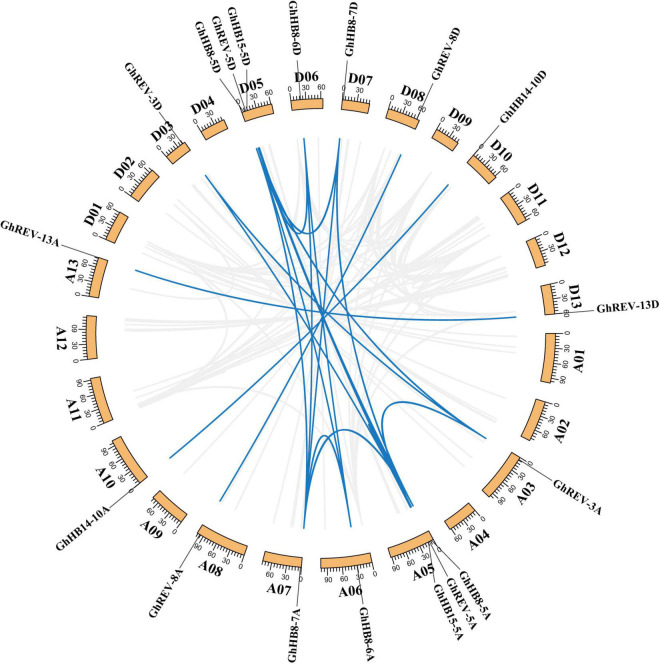
Chromosomal distribution and gene duplication of *HD-ZIP III* genes in cotton (*Gossypium hirsutum*). The value on each chromosome represents chromosome length. Blue lines indicate duplicated *HD-ZIP III* gene pairs.

The physical location of cotton *HD-ZIP IIIs* on chromosomes was determined using the chromosomal location information extracted from the cotton genome database (Supplementary Figure 1), and it was found that the 18 *HD-ZIP IIIs* were unevenly distributed on different chromosomes but symmetrically in A and D-subgenomes. Chromosome 5 contains most, i.e., six HD-ZIP III genes, while the remaining 12 genes are evenly distributed on six other chromosomes. A total of 23 pairs of collinearity genes were detected, all of which were located on different chromosomes (Figure 3), suggesting that gene segmental duplication may be the primary route of HD-ZIP III subfamily expansion and evolution.

**FIGURE 4:**
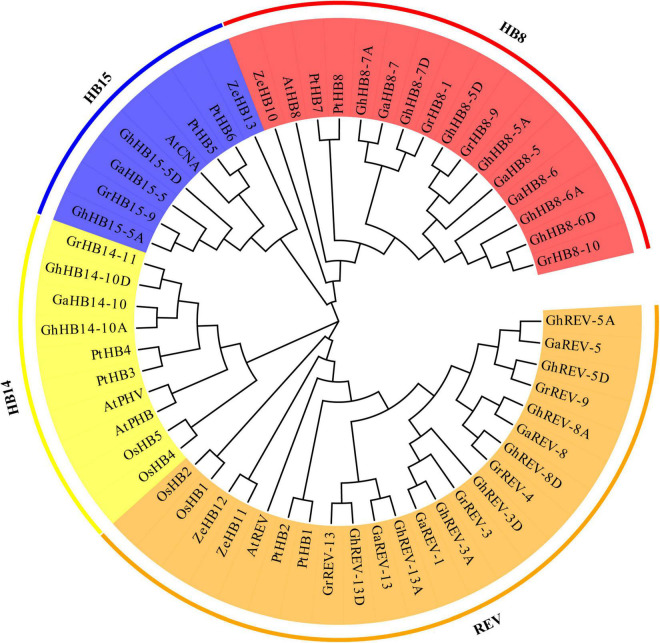
Phylogenetic tree of *Arabidopsis*, *Gossypium arboretum*, *Gossypium hirsutum*, *Gossypium raimondii*, *Oryza Sativa*, *Populus trichocarpa*, and *Zinnia elegans* HD-ZIP III proteins. All the protein sequences were aligned by ClustalX and the phylogenetic tree was constructed by MEGA7 using the maximum likelihood method with 1000 bootstrap replications. The four groups are represented with different colors.

To understand the evolutionary relationship of cotton *HD-ZIP III* genes and their orthologs in different plant species, we construct a phylogenetic tree from the alignment of 18 *G. hirsutum*, 9 *G. arboretum*, 9 *G. raimondii*, 5 *A. thaliana*, 5 *O. sativa*, 8 *P. trichocarpa*, 4 *Z. elegans* HD-ZIP III protein sequences (Supplementary Table 3). As shown in Figure 4, the HD-ZIP III subfamily can be divided into four groups: REV, HB8, HB14, and HB15. The members of *G. hirsutum*, *G. arboretum*, *G. raimondii*, *A. thaliana*, and *P. trichocarpa* HD-ZIP IIIs were dispersed in each group, whereas the *O. sativa* homolog was not present in the HB8 group and the *Z. elegans* homolog was not included in the group HB14, suggestive of lineage-specific gene loss in these two species. Except for HB14, single *REV*, *AtHB8*, and *CNA* genes were found in the subclades of REV, HB8, and HB15 in Arabidopsis, whereas multiple genes were found in tetraploid *G. hirsutum*, which may indicate that the duplication of *HD-ZIP III* genes had occurred in *G. hirsutum* after the divergence of *G. hirsutum* and Arabidopsis.

### Expression Patterns of *HD-ZIP III* Members in Different Cotton Tissues

**FIGURE 5:**
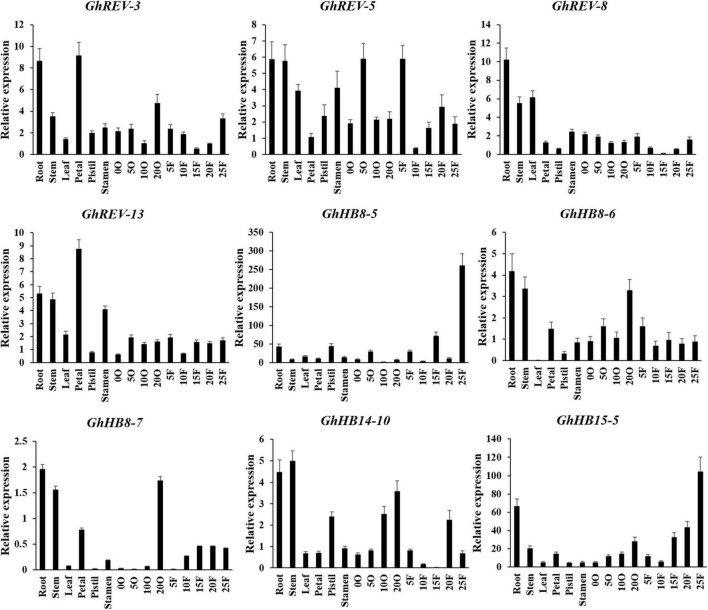
Expression profiles of cotton *HD-ZIP III* genes in different cotton tissues. *GhUBI1* was used as the internal control for normalization. Values represent the mean ± SD of three biological replicates. 0O, ovule in anthesis; 5O, ovules in 5 days post anthesis (DPA); 10O, ovules in 10 DPA; 20O, ovules in 20 DPA; 5F, fibers in 5 DPA; 10F, fibers in 10 DPA; 15F, fibers in 15 DPA; 20F, fibers in 20 DPA; 25F, fibers in 25 DPA.

To get insight into the expression profiles of the cotton HD-ZIP III subfamily, we performed qRT-PCR using the RNAs derived from various cotton tissues. Because the sequences of two homologous genes from the A and D subgenome are very similar, we designed a pair of consensus primers for the two genes (Supplementary Table 5). It was shown that *GhREV-3*, *5*, *8*, and *13* were highly expressed in both vegetative and reproductive organs, whereas *GhHB8-6*, *7* and *GhHB14-10* were mainly expressed in root, stem, and 20 days post anthesis (DPA) ovule. *GhHB8-5* and *GhHB15-5* were expressed preferentially in 25 DPA fibers (Figure 5). The diversified expression patterns of *HD-ZIP III* genes in cotton may indicate their functional diversity and versatility relevant to cotton growth and development.

In order to further clarify the expression of *GhHB8-5A* and *5D*, the two homoeologous genes of *GhHB8-5*, first, we compared the *cis*-elements in the promoter regions of *GhHB8-5A* and *5D*, and their variations were documented in Supplementary Table 4. Then, we designed primers based on the most variable regions in order to differentiate the homoeologous genes (Supplementary Table 5) for qRT-PCR. As shown in Supplementary Figure 2, the expression of *GhHB8-5A* was abundant in root, flower, 20 and 25 DPA fiber. *GhHB8-5D* was predominantly expressed in fiber, and its expression level increased along with the progress of fiber development. Hence, *GhHB8-5D* might be a key player in fiber development and was selected for further study. In addition, we found that *GhHB8-5A* and *5D* displayed similar expression patterns during fiber development, which suggested that there may be functional redundancy between them.

### Transcription Factor Characteristic Analysis of GhHB8-5D

**FIGURE 6:**
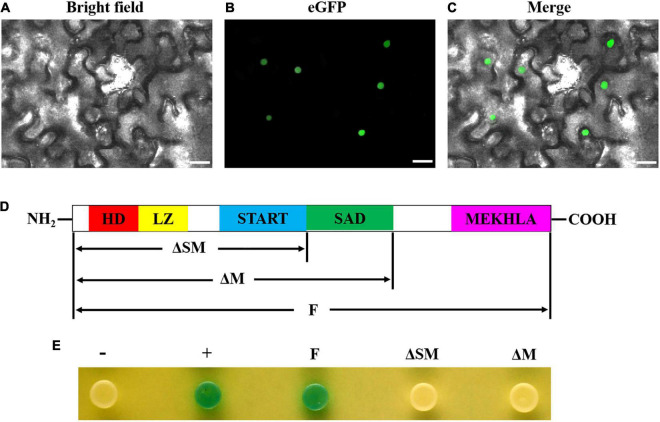
GhHB8-5D is a typical transcriptional activator. **(A–C)** The subcellular location of GhHB8-5D in epidermal cells of tobacco leaves. Bars = 50 μm. **(D)** Schematic diagram shows domain constructs of GhHB8-5D. **(E)** Assay of GhHB8-5D transcriptional activation activity in yeast. –, negative control; +, positive control; F, full-length of GhHB8-5D; ΔSM, a truncated version of GhHB8-5D lacking SAD and MEKHLA domains; ΔM, a truncated version of GhHB8-5D lacking MEKHLA domain.

To further determine whether GhHB8-5D is a transcriptional activator, we transiently expressed GhHB8-5D-eGFP fusion protein in *N. benthamiana* leaf cells, which demonstrated that GFP signals were only detectable in the nucleus (Figures 6A–C). Additionally, various versions of *GhHB8-5D*, including full-length (F), both SAD- and MEKHLA-domain-truncated (ΔSM) and MEKHLA domain-truncated (ΔM), were individually inserted into the pGBKT7 vector and expressed in yeast strain AH109. Empty pGBKT7 vector was used as a negative control. The transformed yeast cells all grew well on the selective SD/-Trp medium, but only positive control and transformed yeast cells harboring full-length GhHB8-5D turned blue in color in the presence of X-gal, indicating that GhHB8-5D has transcriptional activation activity and the activation domain is located in the MEKHLA domain (Figures 6D,E).

### Expression of *GhHB8-5D* in *Arabidopsis* Altered Xylem Differentiation and Secondary Wall Deposition

**FIGURE 7:**
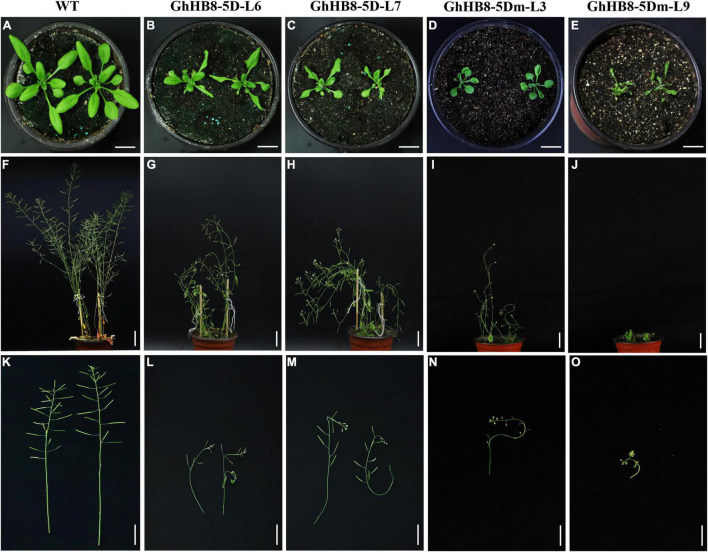
The phenotype of wild type and *GhHB8-5D* and *GhHB8-5Dm* transgenic lines. **(A–E)** Three-week-old seedlings of wild type and transgenic lines. **(F–J)** Six-week-old plants of wild type and transgenic lines. **(K–O)** Magnified images of the inflorescence stem from 6-week-old wild type and transgenic lines. WT represent wild type plants. L6 and L7 indicate two independent lines of *GhHB8-5D* overexpression plants. L3 and L9 denote two independent lines of *GhHB8-5Dm* overexpression plants in which the miR166 binding site was executed synonymous mutation. Bars = 1 cm.

**FIGURE 8:**
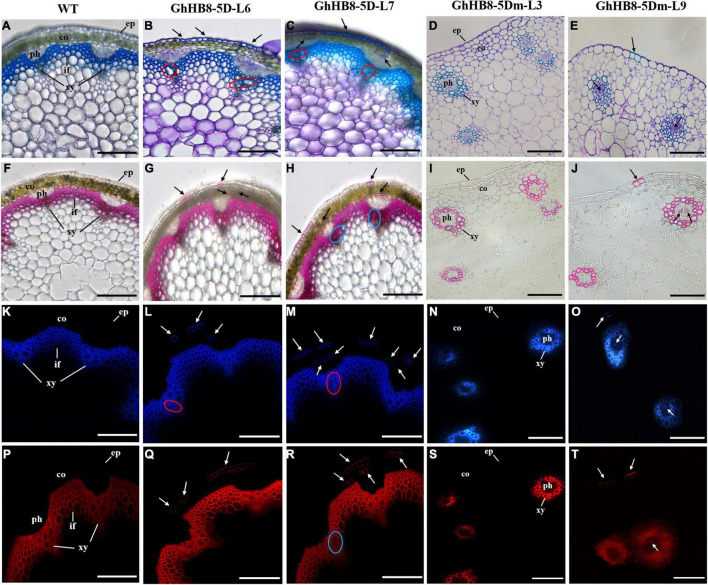
Cross-sections of the 6-week-old stem from wild type and transgenic lines. **(A–E)** Toluidine blue staining of 6-week-old stems from wild type and transgenic lines. The blue regions indicate the deposition of the secondary wall; **(F–J)** phloroglucin staining of 6-week-old stems from wild type and transgenic lines. The red regions indicate the deposition of lignin. **(K–O)** Lignin autofluorescence of 6-week-old stems from wild type and transgenic lines. Regions with blue fluorescence indicate the deposition of lignin. **(P–T)** Pontamine Fast Scarlet 4B (S4B) staining of 6-week-old stems from wild type and transgenic lines. Regions with red fluorescence indicate the deposition of cellulose. Black arrows show ectopic deposition of the secondary wall. Red and blue circles show irregular xylem vessels. ep, epidermis; co, cortex; ph, phloem; xy, xylem; if, interfascicular fiber. Bars = 100 μm.

To further study the physiological function of *GhHB8-5D* and avoid the interference of microRNA to its function, we first aligned the START domain sequences of *GhHB8-5D* and *AtHB8*, which revealed that their microRNA binding sites were identical (Supplementary Figure 3). Then, vectors overexpressing *GhHB8-5D* or *GhHB8-5Dm* (synonymous mutation of the miR binding site, Supplementary Figure 4) were constructed and used to transform Arabidopsis. Transgenic *A. thaliana* plants were verified by using semiquantitative RT-PCR and two independent homozygous lines overexpressing *GhHB8-5D* or *GhHB8-5Dm* were selected for further analyses (Supplementary Figure 5). Compared with the wild type, 3-week-old transgenic seedlings were obviously smaller with curly leaves (Figures 7A–E). Six-week-old transgenic plants were significantly shorter than wild type (Figures 7F–J) and their stems were twisted (Figures 7K–O). Subsequently, cross-sections of the 6-week-old stems from wild type and transgenic plants were stained by using toluidine blue, or phloroglucinol or Pontamine Fast Scarlet 4B (S4B). As shown in Figure 8, Arabidopsis plants overexpressing *GhHB8-5D* displayed ectopic deposition of the secondary wall in some parenchymatous cells (Figures 8B,C) compared with the wild type, in which secondary wall deposition existed only in the xylem and interfascicular fibers (Figure 8A). Staining with phloroglucinol or S4B or lignin autofluorescence revealed that *GhHB8-5D* overexpression caused ectopic deposition of secondary wall components, including lignin and cellulose (Figures 8G,H,L,M,Q,R). It is worthy of noting that some irregular xylem vessels appeared in the *GhHB8-5D* overexpression lines (Figures 8B,C,H,L,M,R). In wild type, xylems and interfascicular fibers were arranged in a ring shape. But such a regular organization was disrupted in the *GhHB8-5Dm* transgenic lines due to the aberrant placement of amphivasal vascular bundles. Moreover, there was no deposition of the secondary wall at the position of interfascicular fibers (Figures 8D,E,I,J,N,O,S,T). Interestingly, a few ectopic depositions of the secondary wall appeared in the epidermis and phloems of the transgenic line #9 expressing *GhHB8-5Dm* (Figures 8E,J,O,T).

**FIGURE 9:**
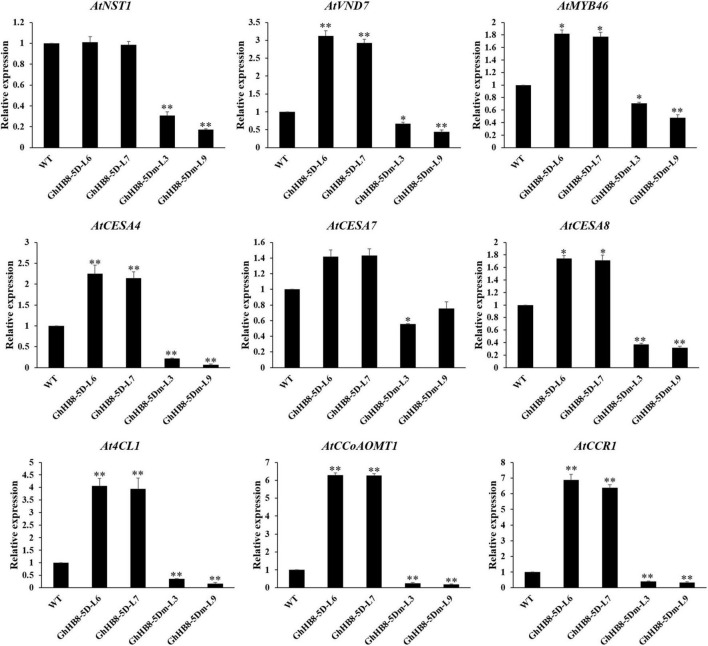
Expression of secondary wall-related genes in 6-week-old stems of wild type and transgenic lines. The *AtActin2* was used as an internal control for normalization. Values represent the mean ± SD of three biological replicates. Student’s *t*-tests demonstrated that there were significant differences (*p < 0.05, ^**^P < 0.01) between the transgenic lines and the wild type.

To better understand the mechanism underlying the ectopic secondary wall deposition as a result of overexpressing *GhHB8-5D*, we investigated whether GhHB8-5D could induce the expression of secondary wall-related genes in transgenic *Arabidopsis*. Consistent with the ectopic deposition of secondary walls, *GhHB8-5D* overexpression led to upregulating the transcription level of most secondary wall biosynthetic genes significantly, except *AtNST1* and *AtCESA7* (Figure 9). However, overexpression of *GhHB8-5Dm* could repress the expression of nine secondary wall-related genes, including three master switches for the biosynthesis of the secondary wall (*AtNST1*, *AtVND7*, and *AtMYB46*), three cellulose synthase genes (*AtCESA4*, *AtCESA7*, and *AtCESA8*), and three lignin biosynthetic genes (*At4CL1*, *AtCCoAOMT1*, and *AtCCR1*). These results indicated that GhHB8-5D may affect the deposition of the secondary wall by regulating the expression of secondary wall-related genes.

As the Arabidopsis *HD-ZIP III* genes were also involved in the morphogenesis of the vascular system, would the overexpressing *GhHB8-5D* influence the xylem morphology by interfering with the expressions of *HD-ZIP III* genes in *Arabidopsis*? In order to answer such a question, we examined the expression levels of *Arabidopsis HD-ZIP III* genes in wild type and transgenic lines. As shown in Supplementary Figure 6, *GhHB8-5D* overexpression led to downregulating the transcription level of *AtHB8* and *AtPHV* but upregulating the transcription level of *AtREV*. Overexpression of *GhHB8-5Dm* could induce the expression of *AtPHB* and *AtPHV* but represses the expression of *AtHB8*, *AtCNA*, and *AtREV*. These results implied that GhHB8-5D may influence the xylem morphology by interfering with the expression of *Arabidopsis HD-ZIP III* genes.

### GhHB8-5D Is Involved in Drought Resistance in the Transgenic Seedlings

**FIGURE 10:**
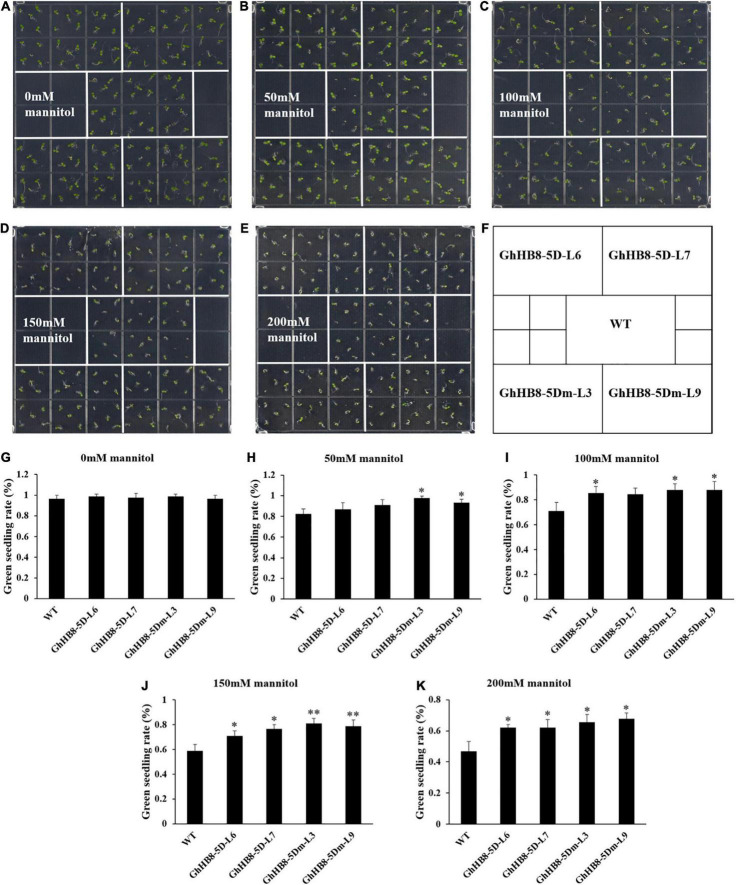
Assay of the green seedling rate of wile type and transgenic lines overexpressing *GhHB8-5D* and *GhHB8-5Dm* under mannitol treatment. **(A–E)** Postgermination green seedling analysis of the wild type and transgenic lines overexpressing *GhHB8-5D* and *GhHB8-5Dm* grown on MS medium supplemented with 0, 50, 100, 150, and 200 mM mannitol for 4 days. **(F)** Schematic diagram of wild type and transgenic seedlings distribution on the MS medium. **(G–K)** Statistical analysis of the green seedling rate of wild type and transgenic seedlings grown on MS medium containing 0, 50, 100, 150, and 200 mM mannitol for 4 days. Mean values and SD are shown from three biological replicates. Independent *t*-tests demonstrate that there are significant (*p* < 0.05) or very significant (*p* < 0.01) differences in green seedling rate between wild type and transgenic lines. *means significant differences (*p* < 0.05); **means very significant differences (*p* < 0.01).

It has been reported that the members of the HD-ZIP III subfamily are associated with water utilization efficiency and abiotic stresses (Xie et al., 2015; Ramachandran et al., 2018; Zhang et al., 2018b). In this study, we have identified a drought-induced *cis*-element in the promoter region of *GhHB8-5D* (Figure 2). To investigate whether GhHB8-5D is related to drought tolerance, we observed seed germination and counted the rate of green seedlings on MS medium supplemented with different concentrations of mannitol to mimic the drought stress. *GhHB8-5D* and *GhHB8-5Dm* overexpression seeds showed normal germination as did wild type seeds under normal conditions. The green seedling rate of both wild type and transgenic lines reached almost 100% after 4 days (Figures 10A,G). When sowing on MS medium containing 50 mM mannitol, green seedling rate of *GhHB8-5D* overexpression lines was slightly higher compared with that of wild type, but the rate of *GhHB8-5Dm* overexpression lines was significantly higher than that of wild type (Figures 10B,H). In the presence of 100 mM mannitol, the green seedling rate was 71.1%, but it was significantly raised in the range of 84.4–87.8% in the transgenic lines (Figures 10C,I). Similarly, four transgenic lines showed an obviously higher green seedling rate on MS medium with 150 mM and 200 mM mannitol, compared with that of wild type in the same conditions (Figures 10D,E,J,K).

**FIGURE 11:**
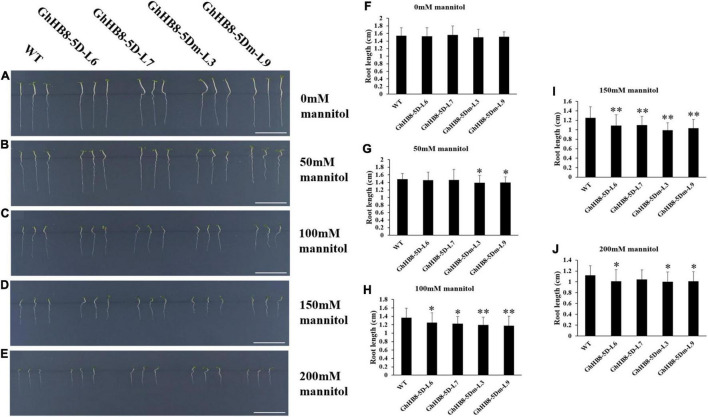
Analysis of root length of wild type and transgenic lines overexpressing *GhHB8-5D* and *GhHB8-5Dm* under mannitol treatment. **(A–E)** The phenotype of wild type and transgenic seedlings under different concentrations of mannitol treatment for 7 days. Bars = 1 cm. **(F–J)** Statistical analysis of root length of wild type and transgenic seedlings under different concentrations of mannitol treatment for 7 days. Mean values and SD are shown from three biological replicates. Independent *t*-tests demonstrate that there are significant (*p* < 0.05) or very significant (*p* < 0.01) differences in root length between wild type and transgenic lines. *means significant differences (*p* < 0.05); **means very significant differences (*p* < 0.01).

In addition, the seeds of wild type and transgenic lines were sowed on MS medium supplemented with 0, 50, 100, 150, and 200 mM mannitol for 7 days prior to the measurement of the primary root lengths. As shown in Figures 11A,F, there was no statistically meaningful difference in the primary root length between wild type and transgenic lines without mannitol treatment. In contrast, in the presence of 50 mM mannitol, the primary root length of transgenic lines overexpressing *GhHB8-5Dm* was significantly shorter than that of wild type (Figures 11B,G). With the increase of mannitol concentration (100, 150, and 200 mM), root growth of both wild type and transgenic lines was suppressed dramatically, and the primary root length of the wild type was remarkably longer than that of transgenic lines (Figures 11C–E,H–J). These results indicated that *GhHB8-5D* may be induced by drought and involved in drought resistance.

## Discussion

HD-ZIP III belongs to a plant-specific and highly conserved protein subfamily, the members of which play vital roles in plant differentiation and development. Although HD-ZIP III has been studied in several plants (Chai et al., 2018; Li et al., 2019; Sharif et al., 2020), this study represents the first comprehensive investigation on this subfamily of genes in cotton. In this study, we identified 18 *HD-ZIP III* genes in the tetraploid upland cotton genome, which could be divided into four distinct groups. HD-ZIP III and HD-ZIP IV subfamilies share a similar domain arrangement but HD-ZIP IV lacks the MEKHLA domain, suggesting their common origin and relatively recent divergence from a common lineage (Schrick et al., 2004). However, HD-ZIP I and HD-ZIP II only have the HD and LZ domains with spacing different from those of HD-ZIP III and HD-ZIP IV, indicating that their juxtaposition may have evolved independently (Sessa et al., 1998).

The result of the *cis*-element analysis showed that the members of cotton HD-ZIP III may be involved in phytohormone response, abiotic stresses tolerance, phenylpropane metabolism, and plant differentiation and development (Figure 2). It has been reported that phytohormones can regulate the expression of NAC and MYB TFs, which enables them to play a role in secondary wall formation. The application of cytokinin was shown to inhibit the expression of *VND6* and *VND7*, whereas auxin inhibited *VND6* alone. However, when both hormones were applied together, they promoted the expression of both *VND*s. Similarly, complex effects were obtained with the combination of auxin, cytokinin, and brassinolide. The application of brassinolide alone upregulated *VND6*, while brassinolide in combination with auxin had no effect on the expression of *VND*s. The combination of cytokinin and brassinolide caused the transcriptional repression of *VND6* and *VND7*, whereas a mixture of auxin, cytokinin, and brassinolide promoted the expression of these *VND*s (Kubo et al., 2005). The auxin treatment was also shown to downregulate *MYB26* expression and suppress precocious lignification, as demonstrated by the *afb1/myb26* double mutant, which failed to show lignification, manifesting that auxin may act as a negative regulator of lignification *via* the downregulation of *MYB26* (Cecchetti et al., 2013). In addition, abiotic stresses have been found to be responsible for increasing secondary wall substances, especially lignin (Lee et al., 2007), which not only provide terrestrial plants with rigidity against compressive forces but also form a mechanical barrier against drought stress (Moura et al., 2010). The aromatic properties of lignin make the secondary wall impermeable to water, which reduces transpiration and assists with maintaining normal turgor pressures under drought stress (Yao et al., 2021). Therefore, it is conceivable that lignification represents an initial form of protection against drought stress. Several studies have shown that the process of lignification is important for drought tolerance. Overexpression of *IbLEA14* in sweet potato (Park et al., 2011), *OsTF1L* in rice (Bang et al., 2019), *VlbZIP30* in grapevine (Tu et al., 2020), and *PoCCoAOMT* in tobacco (Zhao et al., 2021) all enhanced lignin biosynthesis and drought resistance. In maize, drought-tolerant inbred lines showed higher lignification than drought-sensitive lines, suggesting that lignification is an important adaptation to drought stress (Hu et al., 2009).

Gene duplication is a primary source of “raw material” for evolutionary innovations because redundant paralogs have fewer selective constrains and are ready to evolve new functions than non-redundant genes (Prigge and Clark, 2006). Collinearity analysis of the HD-ZIP III subfamily revealed that all HD-ZIP III members have undergone segmental duplication (Figure 3). This may be the reason for functional redundancy and diversity among HD-ZIP III proteins. Phylogenetic analysis using *HD-ZIP III* homologs from diverse plant species identified three divergent clades that occurred during plant evolution and only one monocot *HD-ZIP III* gene (*OsHB3*) appeared in the HB8/HB15 clade (Figure 4). The three clades of *HD-ZIP III* members were consistent with the analysis of monocot- and eudicot-derived genes indicating that the genome of the ancestral angiosperm plant contained *REV*, *HB14*, and *HB8*/*HB15* paralogs. The relationship of monocot *HD-ZIP III* to the eudicot *HB8*/*HB15* clade suggested that the HB8/HB15 gene duplication event may have taken place after the monocot-eudicot split (Prigge and Clark, 2006). Additionally, most homologs of cotton *HD-ZIP III*s were related to xylem differentiation and secondary wall formation in their respective species (Ohashi-Ito et al., 2002; Ko et al., 2006; Zhu et al., 2013; Du et al., 2015; Zhang et al., 2018b; Chen et al., 2021). Considering the conservation of this subfamily in different plants, we inferred that the genes of the same subfamily in cotton (*Gossypium hirsutum*) may have similar functions.

*GhHB8-5* showed D-subgenome biased expression, possibly due to the promoter difference, as we compared the promoter sequences of *GhHB8-5A* and *5D* and identified variations in their *cis*-elements (Supplementary Table 4). In addition, the GhmiR166 binding site can be found in the START domain of both *GhHB8-5A* and *5D*. The different cleavage efficiency of GhmiR166 to *GhHB8-5A* and *5D* may also lead to their differential expression.

Specific to the influence on the vasculature and cell wall, the HD-ZIP III subfamily can not only change the vascular patterning and organization but also affect the xylem differentiation, which leads to abnormal secondary wall deposition. Overexpression of *AtHB8* in Arabidopsis caused rolled-up leaves, a strong reduction of inflorescence stem elongation, and shorter plant height. The anatomical structure of transgenic plant stem showed an increase in the production of phloem fiber sclereids and the lignified tissues (Baima et al., 2001), which were similar to the phenotype of ectopic expression of *GhHB8-5D* in Arabidopsis, as illustrated in this study (Figures 7, 8). Furthermore, several genes that are known to play significant roles during xylem differentiation and secondary wall formation were upregulated as a result of *PtrHB7* (homolog of *AtHB8*) overexpression in *Populus* and *Arabidopsis* (Zhu et al., 2013). Similarly, ectopic expression of *GhHB8-5D* in Arabidopsis also increased the transcription levels of secondary wall-related TFs, cellulose synthase genes, and lignin biosynthetic genes (Figure 9). Taken together, our results indicated that GhHB8-5D is a positive regulator of xylem differentiation and secondary wall biosynthesis.

However, with the emergence of new reports, the idea that AtHB8 is a negative regulator of xylem differentiation and secondary wall formation was supported by empirical observation. In the process of vascular bundles formation, REV played a role in promoting xylem differentiation and secondary wall deposition. While xylem vessels were present in *rev* mutants, xylary fibers were reduced in weaker alleles and absent in strong alleles. This loss of fibers resulted in large reductions of the secondary wall (Zhong et al., 1997; Zhong and Ye, 1999). Genetic analysis has also supported the above conclusion. REV can bind to the promoter of *VND7*, a master regulator for xylem and secondary wall formation and promote its activity (Endo et al., 2015). Studies on the genetic redundancy between *REV* and the other *HD-ZIP III* TFs showed that *phb* and *phv* are strong enhancers of the *rev* phenotype in the xylem. In contrast, the defects of *rev* mutant were restored in *anth8 cna rev* triple mutants. The overexpression of *AtHB8* and *CNA* driven by *REV* promoter in the *rev* mutant background was not able to rescue the phenotypic defects of *rev*. Taken together, these observations suggested that *PHB* and *PHV* genes performed overlapping functions with *REV*, but *AtHB8* and *CNA* played roles antagonistic to *REV* in xylem differentiation (Prigge et al., 2005). In addition, the phenotypes of gain-of-function *phb-1d* and *phv-1d* showed amphivasal vascular bundles with xylem surrounding phloem (McConnell et al., 2001). In transgenic Arabidopsis lines overexpressing *GhHB8-5Dm*, the expression levels of *AtPHB* and *AtPHV* were significantly higher than those in wild type (Supplementary Figure 6). This may explain why amphivasal vascular bundles appeared in these transgenic plants (Figure 8). Under such phenotypic changes, there must be a sophisticated regulatory network. *ACL5* (*ACAULIS 5*) is a gene encoding a thermospermine synthase, which has been shown to regulate xylem differentiation and secondary wall deposition in a negative manner (Muñiz et al., 2008). AtHB8 acts together with auxin as a direct positive regulator of *ACL5*, which slows xylem differentiation and secondary wall formation, in part by negative regulation of *REV* (Baima et al., 2014). This may explain the altered expression patterns of those genes involved in secondary wall-related genes, as a result of overexpressing *GhHB8-5D* and *GhHB8-5Dm* in Arabidopsis (Figure 9).

Recent reports have revealed that the HD-ZIP III subfamily participated in the regulation of response to drought stress. Rice plants overexpressing *OsHB4*, a member of the HD-ZIP III subfamily and a major target of miR166, resembled the phenotypes of the miR166 knockdown plants showing enhanced drought resistance (Zhang et al., 2018b). The suppression of miR166 was found to be coincidental with the upregulation of *HD-ZIP III* as a result of drought treatment (Xie et al., 2015). Further, water-limiting conditions caused major changes to the root xylem morphology (Ramachandran et al., 2018). Similar changes have also been observed in poplar and soybean, where drought stress resulted in an increase in vessel number and vessel wall thickness, thereby conferring resistance to water deficiency (Awad et al., 2010; Prince et al., 2017). Using the combination of mannitol treatment and statistical analysis, we showed that transgenic Arabidopsis overexpressing *GhHB8-5D* and *GhHB8-5Dm* had a higher green seedling rate but shorter root length compared with wild type (Figures 10, 11). GhHB8-5D can affect xylem morphology and secondary wall biosynthesis and could, therefore, be responsible for the improvement in drought resistance and alteration in root length in transgenic plants.

In this study, we comprehensively analyzed the conservative domains, *cis*-elements contained in the promoter regions, location and duplication, evolutionary relationship, and expression of *HD-ZIP III* subfamily in cotton. Furthermore, we established GhHB8-5D as a transcriptional activator and its C-terminus as the transactivation domain. GhHB8-5D could be involved in drought resistance by influencing xylem morphology and secondary wall biosynthesis. Our findings may provide a novel strategy for improving plant adaptations to environmental changes *via* regulating plant cell wall synthesis.

## Data Availability Statement

The datasets presented in this study can be found in online repositories. The names of the repository/repositories and accession number(s) can be found in the article/Supplementary Material.

## Author Contributions

WX and FL designed the experiments. JZ, YG, and MF performed the experiments. JZ, YC, SL, and LL analyzed the data. YW prepared the plant materials. JZ, WX, and FL wrote and revised the manuscript. All authors contributed to the article and approved the submitted version.

## Conflict of Interest

The authors declare that the research was conducted in the absence of any commercial or financial relationships that could be construed as a potential conflict of interest.

## Publisher’s Note

All claims expressed in this article are solely those of the authors and do not necessarily represent those of their affiliated organizations, or those of the publisher, the editors and the reviewers. Any product that may be evaluated in this article, or claim that may be made by its manufacturer, is not guaranteed or endorsed by the publisher.

## Footnotes

1
http://www.arabidopsis.org

2
https://www.cottongen.org

3
http://pfam.xfam.org

4
https://www.ebi.ac.uk/Tools/hmmer

5
https://www.ncbi.nlm.nih.gov

6
https://www.expasy.org/resources/protparam

7
http://www.csbio.sjtu.edu.cn/bioinf/plant-multi

8
http://gsds.gao-lab.org

9
https://meme-suite.org/meme/tools/meme

10
http://smart.embl-heidelberg.de/smart/batch.pl

11
http://bioinformatics.psb.ugent.be/webtools/plantcare/html

## References

[B1] Agalou A. Purwantomo S. Overnäs E. Johannesson H. Zhu X. Estiati A. (2008). A genome-wide survey of HD-Zip genes in rice and analysis of drought-responsive family members. *Plant Mol. Biol*. 66 87–103. 10.1007/s11103-007-9255-7 17999151

[B2] Ariel F. D. Manavella P. A. Dezar C. A. Chan R. L. (2007). The true story of the HD-ZIP family. *Trends Plant. Sci*. 12 419–426. 10.1016/j.tplants.2007.08.003 17698401

[B3] Awad H. Barigah T. Badel E. Cochard H. Herbette S. (2010). Poplar vulnerability to xylem cavitation acclimates to drier soil conditions. *Physiol. Plantarum*. 139 280–288. 10.1111/j.1399-3054.2010.01367.x 20210873

[B4] Baima S. Forte V. Possenti M. Peñalosa A. Leoni G. Salvi S. (2014). Negative feedback regulation of auxin signaling by ATHB8/ACL5-BUD2 transcription module. *Mol. Plant* 7 1006–1025. 10.1093/mp/ssu051 24777988

[B5] Baima S. Nobili F. Sessa G. Lucchetti S. Ruberti I. Morelli G. (1995). The expression of the Athb-8 homeobox gene is restricted to provascular cells in *Arabidopsis thaliana*. *Development* 121 4171–4182. 10.1111/j.1365-2303.1995.tb00491.x 8575317

[B6] Baima S. Possenti M. Matteucci A. Wisman E. Altamura M. M. Ruberti I. (2001). The Arabidopsis ATHB-8 HD-zip protein acts as a differentiation-promoting transcription factor of the vascular meristems. *Plant Physiol*. 126 643–655. 10.1104/pp.126.2.643 11402194 PMC111156

[B7] Bang S. W. Lee D. K. Jung H. Chung P. J. Kim Y. S. Choi Y. D. (2019). Overexpression of OsTF1L, a rice HD-Zip transcription factor, promotes lignin biosynthesis and stomatal closure that improves drought tolerance. *Plant Biotechnol. J*. 17 118–131. 10.1111/pbi.12951 29781573 PMC6330637

[B8] Cao J. F. Zhao B. Huang C. C. Chen Z. W. Zhao T. Liu H. R. (2020). The miR319-targeted GhTCP4 promotes the transition from cell elongation to wall thickening in cotton fiber. *Mol. Plant*. 13 1063–1077. 10.1016/j.molp.2020.05.006 32422188

[B9] Carlsbecker A. Lee J. Y. Roberts C. J. Dettmer J. Lehesranta S. Zhou J. (2010). Cell signalling by microRNA165/6 directs gene dose-dependent root cell fate. *Nature* 465 316–321. 10.1038/nature08977 20410882 PMC2967782

[B10] Cecchetti V. Altamura M. M. Brunetti P. Petrocelli V. Falasca G. Ljung K. (2013). Auxin controls *Arabidopsis* anther dehiscence by regulating endothecium lignification and jasmonic acid biosynthesis. *Plant J*. 74 411–422. 10.1111/tpj.12130 23410518

[B11] Chai W. Si W. Ji W. Qin Q. Zhao M. Jiang H. (2018). Genome-wide investigation and expression profiling of HD-Zip transcription factors in Foxtail millet (*Setaria italica* L.). *Biomed. Res. Int*. 2018:8457614. 10.1155/2018/8457614 29862293 PMC5976958

[B12] Chen C. Chen H. Zhang Y. Thomas H. R. Frank M. H. He Y. (2020). TBtools: an integrative toolkit developed for interactive analyses of big biological data. *Mol. Plant* 13 1194–1202. 10.1016/j.molp.2020.06.009 32585190

[B13] Chen H. Fang R. Deng R. Li J. (2021). The OsmiRNA166b-OsHox32 pair regulates mechanical strength of rice plants by modulating cell wall biosynthesis. *Plant Biotechnol. J*. 19 1468–1480. 10.1111/pbi.13565 33560572 PMC8313131

[B14] Du J. Miura E. Robischon M. Martinez C. Groover A. (2011). The Populus Class III HD ZIP transcription factor POPCORONA affects cell differentiation during secondary growth of woody stems. *PLoS One* 6:e17458. 10.1371/journal.pone.0017458 21386988 PMC3046250

[B15] Du Q. Avci U. Li S. Gallego-Giraldo L. Pattathil S. Qi L. (2015). Activation of miR165b represses AtHB15 expression and induces pith secondary wall development in *Arabidopsis*. *Plant J*. 83 388–400. 10.1111/tpj.12897 26043238

[B16] Emery J. F. Floyd S. K. Alvarez J. Eshed Y. Hawker N. P. Izhaki A. (2003). Radial patterning of *Arabidopsis* shoots by class III HD-ZIP and KANADI genes. *Curr. Biol*. 13 1768–1774. 10.1016/j.cub.2003.09.035 14561401

[B17] Endo H. Yamaguchi M. Tamura T. Nakano Y. Nishikubo N. Yoneda A. (2015). Multiple classes of transcription factors regulate the expression of VASCULAR-RELATED NAC-DOMAIN7, a master switch of xylem vessel differentiation. *Plant Cell Physiol*. 56 242–254. 10.1093/pcp/pcu134 25265867

[B18] Haigler C. H. Betancur L. Stiff M. R. Tuttle J. R. (2012). Cotton fiber: a powerful single-cell model for cell wall and cellulose research. *Front. Plant Sci*. 3:104. 10.3389/fpls.2012.00104 22661979 PMC3356883

[B19] Hernandez-Gomez M. C. Runavot J. L. Guo X. Bourot S. Benians T. A. Willats W. G. (2015). Heteromannan and heteroxylan cell wall polysaccharides display different dynamics during the elongation and secondary cell wall deposition phases of cotton fiber cell development. *Plant Cell Physiol*. 56 1786–1797. 10.1093/pcp/pcv101 26187898 PMC4562070

[B20] Hu Y. Li W. C. Xu Y. Q. Li G. J. Liao Y. Fu F. L. (2009). Differential expression of candidate genes for lignin biosynthesis under drought stress in maize leaves. *J. Appl. Genet*. 50 213–223. 10.1007/BF03195675 19638676

[B21] Huang J. Chen F. Guo Y. Gan X. Yang M. Zeng W. (2021). GhMYB7 promotes secondary wall cellulose deposition in cotton fibres by regulating GhCesA gene expression through three distinct cis-elements. *New Phytol.* 232, 1718–1737. 10.1111/nph.17612 34245570

[B22] Huang J. Guo Y. Sun Q. Zeng W. Li J. Li X. (2019). Genome-wide identification of R2R3-MYB transcription factors regulating secondary cell wall thickening in cotton fiber development. *Plant Cell Physiol*. 60 687–701. 10.1093/pcp/pcy238 30576529

[B23] Kidner C. A. Martienssen R. A. (2004). Spatially restricted microRNA directs leaf polarity through ARGONAUTE1. *Nature* 428 81–84. 10.1038/nature02366 14999284

[B24] Kim J. Jung J. H. Reyes J. L. Kim Y. S. Kim S. Y. Chung K. S. (2005). microRNA-directed cleavage of ATHB15 mRNA regulates vascular development in *Arabidopsis* inflorescence stems. *Plant J*. 42 84–94. 10.1111/j.1365-313X.2005.02354.x 15773855 PMC1382282

[B25] Ko J. H. Prassinos C. Han K. H. (2006). Developmental and seasonal expression of PtaHB1, a *Populus* gene encoding a class III HD-Zip protein, is closely associated with secondary growth and inversely correlated with the level of microRNA (miR166). *New Phytol*. 169 469–478. 10.1111/j.1469-8137.2005.01623.x 16411950

[B26] Krzywinski M. Schein J. Birol I. Connors J. Gascoyne R. Horsman D. (2009). Circos: an information aesthetic for comparative genomics. *Genome Res*. 19 1639–1645. 10.1101/gr.092759.109 19541911 PMC2752132

[B27] Kubo M. Udagawa M. Nishikubo N. Horiguchi G. Yamaguchi M. Ito J. (2005). Transcription switches for protoxylem and metaxylem vessel formation. *Genes Dev*. 19 1855–1860. 10.1101/gad.1331305 16103214 PMC1186185

[B28] Kumar S. Stecher G. Tamura K. (2016). MEGA7: molecular evolutionary genetics analysis version 7.0 for bigger datasets. *Mol. Biol. Evol*. 33 1870–1874. 10.1093/molbev/msw054 27004904 PMC8210823

[B29] Larkin M. A. Blackshields G. Brown N. P. Chenna R. McGettigan P. A. McWilliam H. (2007). Clustal W and Clustal X version 2.0. *Bioinformatics* 23 2947–2948. 10.1093/bioinformatics/btm404 17846036

[B30] Lee B. R. Kim K. Y. Jung W. J. Avice J. C. Ourry A. Kim T. H. (2007). Peroxidases and lignification in relation to the intensity of water-deficit stress in white clover (*Trifolium repens* L.). *J. Exp. Bot*. 58 1271–1279. 10.1093/jxb/erl280 17298963

[B31] Li F. Fan G. Lu C. Xiao G. Zou C. Kohel R. J. (2015). Genome sequence of cultivated Upland cotton (*Gossypium hirsutum* TM-1) provides insights into genome evolution. *Nat. Biotechnol*. 33 524–530. 10.1038/nbt.3208 25893780

[B32] Li L. Zheng T. Zhuo X. Li S. Qiu L. Wang J. (2019). Genome-wide identification, characterization and expression analysis of the HD-Zip gene family in the stem development of the woody plant *Prunus mume*. *PeerJ* 7:e7499. 10.7717/peerj.7499 31410318 PMC6689393

[B33] Li Y. Y. Shen A. Xiong W. Sun Q. L. Luo Q. Song T. (2016). Overexpression of OsHox32 results in pleiotropic effects on plant type architecture and leaf development in rice. *Rice* 9:46. 10.1186/s12284-016-0118-1 27624698 PMC5021653

[B34] Liu Y. You S. Taylor-Teeples M. Li W. L. Schuetz M. Brady S. M. (2014). Bel1-like homeodomain6 and knotted arabidopsis thaliana7 interact and regulate secondary cell wall formation via repression of Revoluta. *Plant Cell* 26 4843–4861. 10.1105/tpc.114.128322 25490916 PMC4311193

[B35] McConnell J. R. Emery J. Eshed Y. Bao N. Bowman J. Barton M. K. (2001). Role of PHABULOSA and PHAVOLUTA in determining radial patterning in shoots. *Nature* 411 709–713. 10.1038/35079635 11395776

[B36] McFarlane H. E. Döring A. Persson S. (2014). The cell biology of cellulose synthesis. *Annu. Rev. Plant Biol*. 65 69–94. 10.1146/annurev-arplant-050213-040240 24579997

[B37] Moura J. C. Bonine C. A. de Oliveira Fernandes Viana J. Dornelas M. C. Mazzafera P. (2010). Abiotic and biotic stresses and changes in the lignin content and composition in plants. *J. Integr. Plant Biol*. 52 360–376. 10.1111/j.1744-7909.2010.00892.x 20377698

[B38] Mukherjee K. Burglin T. R. (2006). MEKHLA, a novel domain with similarity to PAS domains, is fused to plant homeodomain-leucine zipper III proteins. *Plant Physiol*. 140 1142–1150. 10.1104/pp.105.073833 16607028 PMC1435804

[B39] Muñiz L. Minguet E. G. Singh S. K. Pesquet E. Vera-Sirera F. Moreau-Courtois C. L. (2008). ACAULIS5 controls *Arabidopsis* xylem specification through the prevention of premature cell death. *Development* 135 2573–2582. 10.1242/dev.019349 18599510

[B40] Ohashi-Ito K. Demura T. Fukuda H. (2002). Promotion of transcript accumulation of novel Zinnia immature xylem-specific HD-Zip III homeobox genes by brassinosteroids. *Plant Cell Physiol*. 43 1146–1153. 10.1093/pcp/pcf135 12407194

[B41] Park S. C. Kim Y. H. Jeong J. C. Kim C. Y. Lee H. S. Bang J. W. (2011). Sweetpotato late embryogenesis abundant 14 (IbLEA14) gene influences lignification and increases osmotic- and salt stress-tolerance of transgenic calli. *Planta* 233 621–634. 10.1007/s00425-010-1326-3 21136074

[B42] Paterson A. H. Wendel J. F. Gundlach H. Guo H. Jenkins J. Jin D. (2012). Repeated polyploidization of *Gossypium* genomes and the evolution of spinnable cotton fibres. *Nature* 492 423–427. 10.1038/nature11798 23257886

[B43] Prigge M. J. Clark S. E. (2006). Evolution of the class III HD-Zip gene family in land plants. *Evol. Dev*. 8 350–361. 10.1111/j.1525-142X.2006.00107.x 16805899

[B44] Prigge M. J. Otsuga D. Alonso J. M. Ecker J. R. Drews G. N. Clark S. E. (2005). Class III homeodomain-leucine zipper gene family members have overlapping, antagonistic, and distinct roles in *Arabidopsis* development. *Plant Cell*. 17 61–76. 10.1105/tpc.104.026161 15598805 PMC544490

[B45] Prince S. J. Murphy M. Mutava R. N. Durnell L. A. Nguyen H. T. (2017). Root xylem plasticity to improve water use and yield in water-stressed soybean. *J. Exp. Bot*. 68 2027–2036. 10.1093/jxb/erw472 28064176 PMC5428998

[B46] Ramachandran P. Wang G. Augstein F. Vries J. D. Carlsbecker A. (2018). Continuous root xylem formation and vascular acclimation to water deficit involves endodermal ABA signalling via miR165. *Development* 145:dev159202. 10.1242/dev.159202 29361572

[B47] Robischon M. Du J. Miura E. Groover A. (2011). The *Populus* Class III HD ZIP, popREVOLUTA, influences cambium initiation and patterning of woody stems. *Plant Physiol*. 155 1214–1225. 10.1104/pp.110.167007 21205615 PMC3046580

[B48] Schena M. Davis R. W. (1992). HD-Zip proteins: members of an *Arabidopsis* homeodomain protein superfamily. *Proc. Natl. Acad. Sci. USA* 89 3894–3898. 10.1073/pnas.89.9.3894 1349174 PMC525597

[B49] Schrick K. Nguyen D. Karlowski W. M. Mayer K. F. (2004). START lipid/sterol-binding domains are amplified in plants and are predominantly associated with homeodomain transcription factors. *Genome Biol*. 5:R41. 10.1186/gb-2004-5-6-r41 15186492 PMC463074

[B50] Sessa G. Steindler C. Morelli G. Ruberti I. (1998). The *Arabidopsis* Athb-8, -9 and -14 genes are members of a small gene family coding for highly related HD-ZIP proteins. *Plant Mol. Biol*. 38 609–622. 10.1023/a:1006016319613 9747806

[B51] Sharif R. Xie C. Wang J. Cao Z. Zhang H. Chen P. (2020). Genome wide identification, characterization and expression analysis of HD-ZIP gene family in *Cucumis sativus* L. under biotic and various abiotic stresses. *Int. J. Biol. Macromol*. 158 502–520. 10.1016/j.ijbiomac.2020.04.124 32376256

[B52] Sparkes I. A. Runions J. Kearns A. Hawes C. (2006). Rapid, transient expression of fluorescent fusion proteins in tobacco plants and generation of stably transformed plants. *Nat. Protoc*. 1 2019–2025. 10.1038/nprot.2006.286 17487191

[B53] Taylor-Teeples M. Lin L. De Lucas M. Turco G. Toal T. W. Gaudinier A. (2015). An *Arabidopsis* gene regulatory network for secondary cell wall synthesis. *Nature* 517 571–575. 10.1038/nature14099 25533953 PMC4333722

[B54] Tu M. Wang X. Yin W. Wang Y. Li Y. Zhang G. (2020). Grapevine VlbZIP30 improves drought resistance by directly activating VvNAC17 and promoting lignin biosynthesis through the regulation of three peroxidase genes. *Hortic. Res*. 7:150. 10.1038/s41438-020-00372-3 32922822 PMC7458916

[B55] Wang T. T. Yu T. F. Fu J. D. Su H. G. Chen J. Zhou Y. B. (2020). Genome-wide analysis of the GRAS gene family and functional identification of GmGRAS37 in drought and salt tolerance. *Front Plant Sci*. 11:604690. 10.3389/fpls.2020.604690 33424904 PMC7793673

[B56] Wang Y. Tang H. Debarry J. D. Tan X. Li J. Wang X. (2012). MCScanX: a toolkit for detection and evolutionary analysis of gene synteny and collinearity. *Nucleic Acids Res*. 40:e49. 10.1093/nar/gkr1293 22217600 PMC3326336

[B57] Xie F. Wang Q. Sun R. Zhang B. (2015). Deep sequencing reveals important roles of microRNAs in response to drought and salinity stress in cotton. *J. Exp. Bot*. 66 789–804. 10.1093/jxb/eru437 25371507 PMC4321542

[B58] Xu W. L. Zhang D. J. Wu Y. F. Qin L. X. Huang G. Q. Li J. (2013). Cotton PRP5 gene encoding a proline-rich protein is involved in fiber development. *Plant Mol. Biol*. 82 353–365. 10.1007/s11103-013-0066-8 23625445

[B59] Yao T. Feng K. Xie M. Barros J. Tschaplinski T. J. Tuskan G. A. (2021). Phylogenetic occurrence of the phenylpropanoid pathway and lignin biosynthesis in plants. *Front. Plant Sci*. 12:704697. 10.3389/fpls.2021.704697 34484267 PMC8416159

[B60] Zhang J. Huang G. Q. Zou D. Yan J. Q. Li Y. Hu S. (2018a). The cotton (*Gossypium hirsutum*) NAC transcription factor (FSN1) as a positive regulator participates in controlling secondary cell wall biosynthesis and modification of fibers. *New Phytol*. 217 625–640. 10.1111/nph.14864 29105766

[B61] Zhang J. Zhang H. Srivastava A. K. Pan Y. Bai J. Fang J. (2018b). Knockdown of rice microRNA166 confers drought resistance by causing leaf rolling and altering stem xylem development. *Plant Physiol*. 176 2082–2094. 10.1104/pp.17.01432 29367235 PMC5841683

[B62] Zhang J. B. Wang X. P. Wang Y. C. Chen Y. H. Luo J. W. Li D. D. (2020). Genome-wide identification and functional characterization of cotton (*Gossypium hirsutum*) MAPKKK gene family in response to drought stress. *BMC Plant Biol*. 20:217. 10.1186/s12870-020-02431-2 32410659 PMC7227315

[B63] Zhang X. Henriques R. Lin S. S. Niu Q. W. Chua N. H. (2006). Agrobacterium-mediated transformation of *Arabidopsis thaliana* using the floral dip method. *Nat. Protoc*. 1 641–646. 10.1038/nprot.2006.97 17406292

[B64] Zhao D. Luan Y. Shi W. Zhang X. Meng J. Tao J. (2021). A Paeonia ostii caffeoyl-CoA O-methyltransferase confers drought stress tolerance by promoting lignin synthesis and ROS scavenging. *Plant Sci*. 303:110765. 10.1016/j.plantsci.2020.110765 33487350

[B65] Zhong R. Taylor J. J. Ye Z. H. (1997). Disruption of interfascicular fiber differentiation in an *Arabidopsis* mutant. *Plant Cell*. 9 2159–2170. 10.1105/tpc.9.12.2159 9437861 PMC157065

[B66] Zhong R. Ye Z. H. (1999). IFL1, a gene regulating interfascicular fiber differentiation in *Arabidopsis*, encodes a homeodomain-leucine zipper protein. *Plant Cell* 11 2139–2152. 10.1105/tpc.11.11.2139 10559440 PMC144121

[B67] Zhong R. Ye Z. H. (2015). Secondary cell walls: biosynthesis, pattern deposition and transcriptional regulation. *Plant Cell Physiol*. 56 195–214. 10.1093/pcp/pcu140 25294860

[B68] Zhu Y. Song D. Sun J. Wang X. Li L. (2013). PtrHB7, a class III HD-Zip gene, plays a critical role in regulation of vascular cambium differentiation in *Populus*. *Mol. Plant*. 6 1331–1343. 10.1093/mp/sss164 23288865

