# Supplementary material for: Genome-Wide Identification of the HD-ZIP III Subfamily in Upland Cotton Reveals the Involvement of GhHB8-5D in the Biosynthesis of Secondary Wall in Fiber and Drought Resistance
Source: Front Plant Sci. 2022 Jan 27;12:806195. doi: 10.3389/fpls.2021.806195 (PMC8828970; doi:10.3389/fpls.2021.806195)
Supplement: Supplementary file 1 [file Data_Sheet_1.zip › Supplementary Figure 1-6.docx]

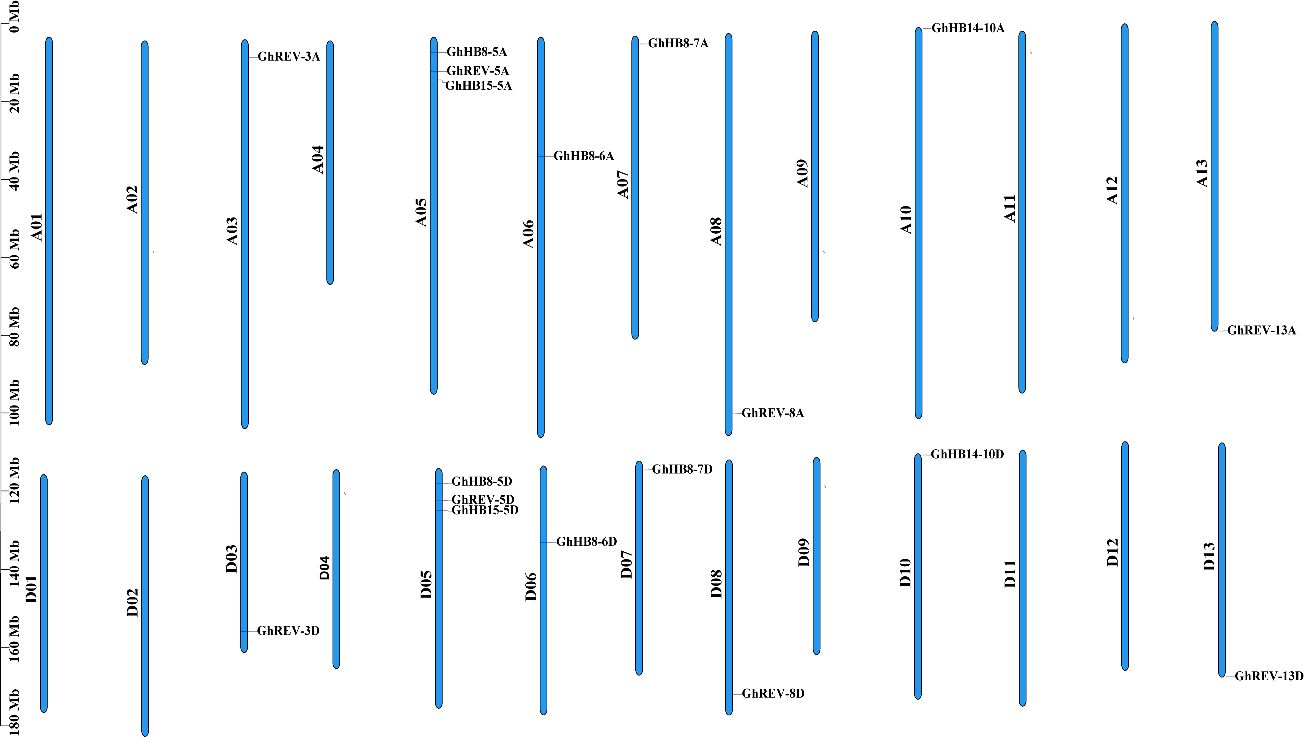


**Supplementary Figure 1**. Chromosomal distribution of 18 *HD-ZIP III* genes in cotton. The scale bar on the left represents the size of cotton chromosomes.


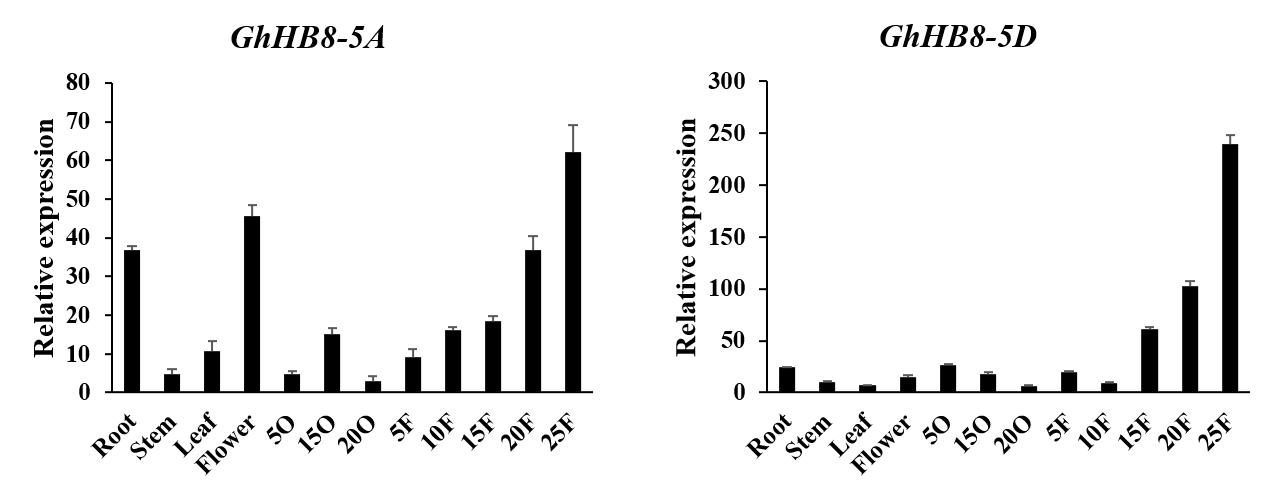


**Supplementary Figure 2**. Expression profiles of *GhHB8-5A* and *GhHB8-5D* in different cotton tissues. *GhUBI1* was used as the internal control for normalization. Values represent mean ± SD of three biological replicates. 0O, ovule in anthesis; 5O, ovules in 5 days post anthesis (DPA); 10O, ovules in 10 DPA; 20O, ovules in 20 DPA; 5F, fibers in 5 DPA; 10F, fibers in 10 DPA; 15F, fibers in 15 DPA; 20F, fibers in 20 DPA; 25F, fibers in 25 DPA.


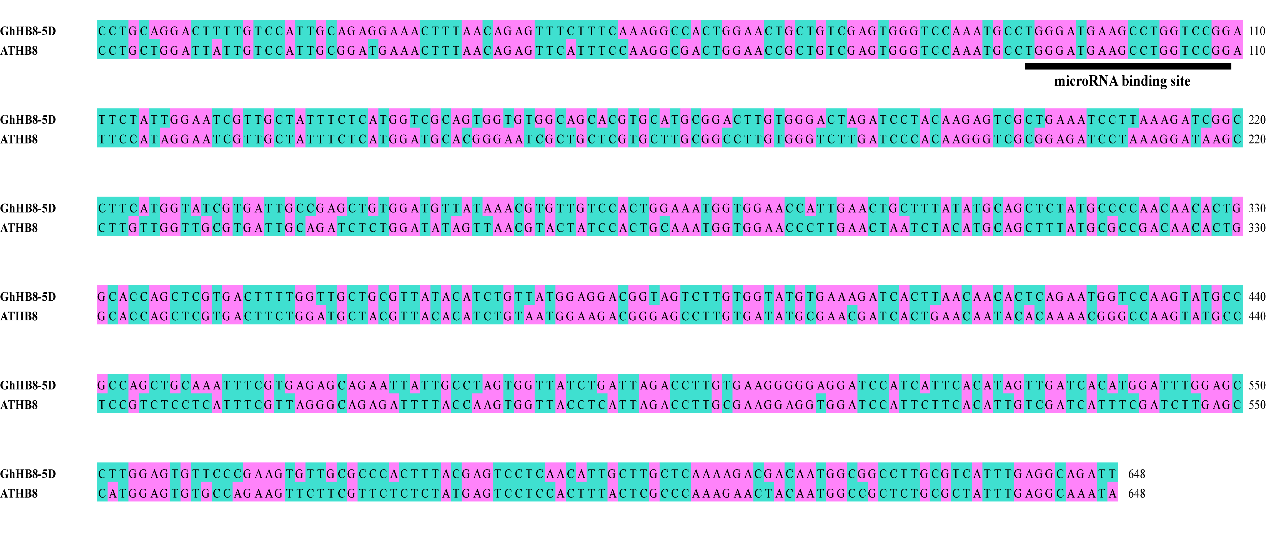


**Supplementary Figure 3**. Alignment of START domain nucleic acid sequence of *GhHB8-5D* and *AtHB8*. Adenine (A) and guanine (G) are in the pink box. Cytosine (C) and thymine (T) are in the cyan box. MicroRNA binding site is shown by underline.


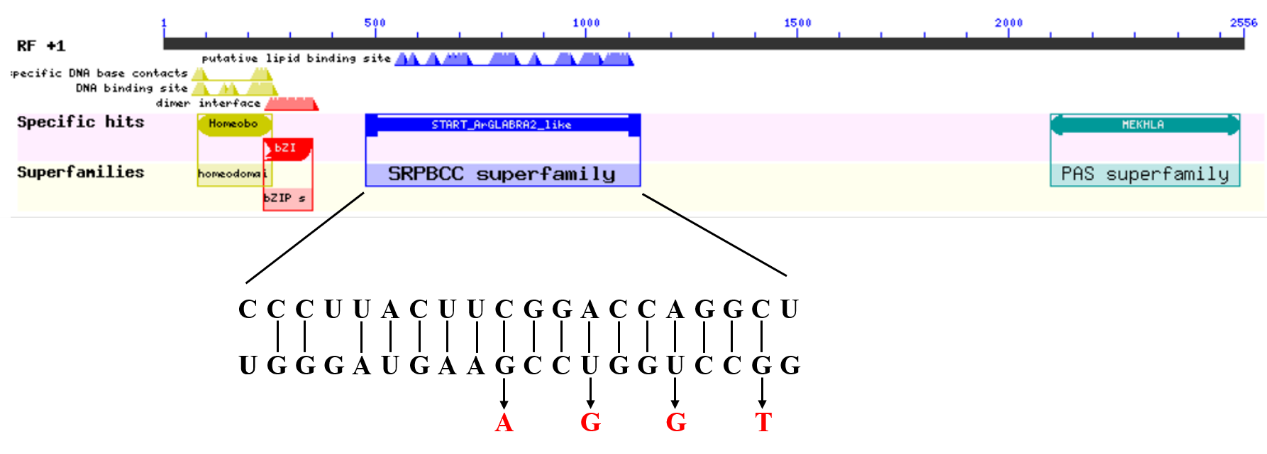


**Supplementary Figure 4**. Schematic diagram of synonymous mutation of miR binding site. Black letters show the original sequences of *miR* and *GhHB8-5D*. Red letters represent synonymous mutated base.


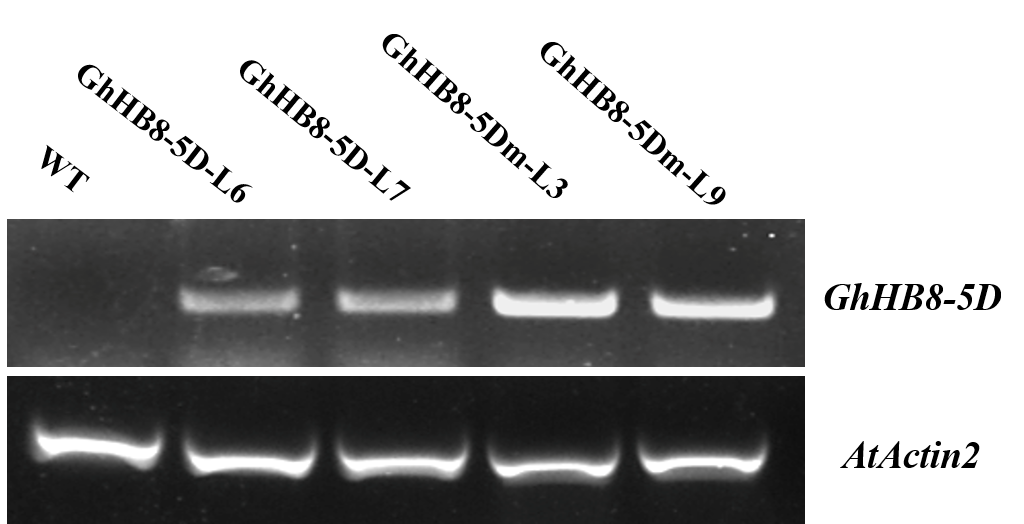


**Supplementary Figure 5**. Semi-quantitative RT-PCR analysis for expression of *GhHB8-5D* in wild type and homozygous transgenic lines. The *AtActin2* is used for normalization.


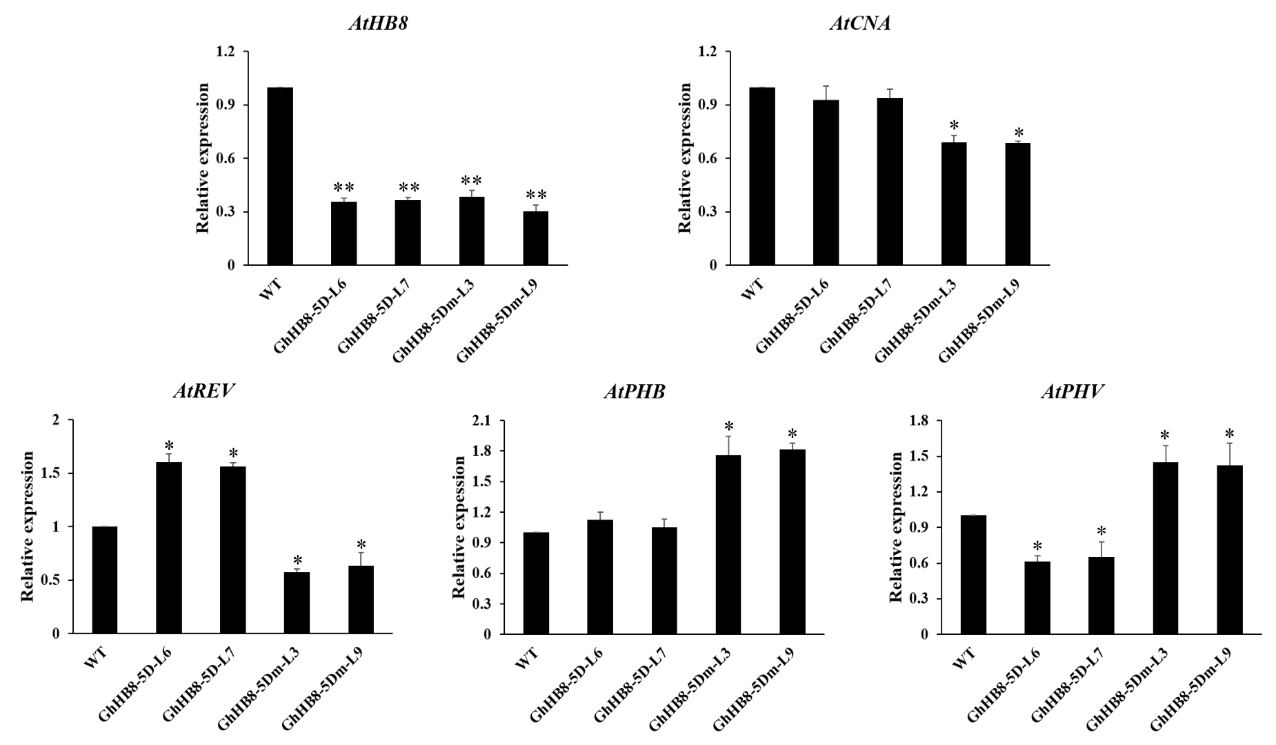


**Supplementary Figure 6**. Expression of *Arabidopsis HD-ZIP III* genes in 6-week-old stems of wild type and transgenic lines. The *AtActin2* was used as an internal control for normalization. Values represent mean ± SD of three biological replicates. Student’s *t*-tests demonstrated that there were significant differences (**p* < 0.05, ** *P* < 0.01) between the transgenic lines and the wild type.
