# Supplementary material for: Genome-Wide Identification of the HD-ZIP III Subfamily in Upland Cotton Reveals the Involvement of GhHB8-5D in the Biosynthesis of Secondary Wall in Fiber and Drought Resistance
Source: Front Plant Sci. 2022 Jan 27;12:806195. doi: 10.3389/fpls.2021.806195 (PMC8828970; doi:10.3389/fpls.2021.806195)
Supplement: Supplementary file 1 [file Data_Sheet_1.zip › Supplementary Table 1.docx]

**Supplementary Table 1. The amino acid sequences of conserved domains of *Gossypium hirsutum* HD-ZIP III subfamily**

**HOMEOBOX domain**

>Gh_A03G0276

E-Value: 1.23E-17 Bitscore: 77.1509

KYVRYTAEQVEALERVYAECPKPSSLRRQQLIRECPILSNIEPKQIKVWFQNRRCREKQ

>Gh_A05G0364

E-Value: 1.71E-18 Bitscore: 79.4621

KYVRYTPEQVEALERLYHECPKPSSMRRQQLIRECPILSNIEPKQIKVWFQNRRCREKQ

>Gh_A05G0892

E-Value: 1.23E-17 Bitscore: 77.1509

KYVRYTAEQVEALERVYAECPKPSSLRRQQLIRECPILSNIEPKQIKVWFQNRRCREKQ

>Gh_A05G1085

E-Value: 7.07E-18 Bitscore: 77.9213

KYVRYTPEQVEALERLYHECPKPSSIRRQQLIRECPILSNIEPKQIKVWFQNRRCREKQ

>Gh_A06G0833

E-Value: 1.84E-18 Bitscore: 79.4621

KYVRYTPEQVEALERLYHECPKPSSMRRQQLIRECPILSNIEPKQIKVWFQNRRCREKQ

>Gh_A07G0163

E-Value: 5.83E-17 Bitscore: 75.2249

KYVRYTPEQVEALERLYYECPKPSSMRRQQLIREYPILSNIEPKQIKVWFQNRRCREKQ

>Gh_A08G1765

E-Value: 2.73E-17 Bitscore: 76.3805

KYVRYTAEQVEALERVYAECPKPSSLRRQQLIRECSILSNIEPKQIKVWFQNRRCRDKQ

>Gh_A10G0035

E-Value: 4.64E-18 Bitscore: 78.3065

KYVRYTPEQVEALERVYNECPKPSSLRRQQLIRECPILSNIEPKQIKVWFQNRRCREKQ

>Gh_A13G2011

E-Value: 1.21E-17 Bitscore: 77.1509

KYVRYTAEQVEALERVYAECPKPSSLRRQQLIRECPILSNIEPKQIKVWFQNRRCREKQ

>Gh_D03G1290

E-Value: 1.24E-17 Bitscore: 77.1509

KYVRYTAEQVEALERVYAECPKPSSLRRQQLIRECPILSNIEPKQIKVWFQNRRCREKQ

>Gh_D05G0479

E-Value: 1.85E-18 Bitscore: 79.4621

KYVRYTPEQVEALERLYHECPKPSSMRRQQLIRECPILSNIEPKQIKVWFQNRRCREKQ

>Gh_D05G0975

E-Value: 1.23E-17 Bitscore: 77.1509

KYVRYTAEQVEALERVYAECPKPSSLRRQQLIRECPILSNIEPKQIKVWFQNRRCREKQ

>Gh_D05G1263

E-Value: 7.10E-18 Bitscore: 77.9213

KYVRYTPEQVEALERLYHECPKPSSIRRQQLIRECPILSNIEPKQIKVWFQNRRCREKQ

>Gh_D06G0966

E-Value: 1.84E-18 Bitscore: 79.4621

KYVRYTPEQVEALERLYHECPKPSSMRRQQLIRECPILSNIEPKQIKVWFQNRRCREKQ

>Gh_D07G0220

E-Value: 5.83E-17 Bitscore: 75.2249

KYVRYTPEQVEALERLYYECPKPSSMRRQQLIREYPILSNIEPKQIKVWFQNRRCREKQ

>Gh_D08G2109

E-Value: 2.73E-17 Bitscore: 76.3805

KYVRYTAEQVEALERVYAECPKPSSLRRQQLIRECPILSNIEPKQIKVWFQNRRCRDKQ

>Gh_D10G0038

E-Value: 4.64E-18 Bitscore: 78.3065

KYVRYTPEQVEALERVYNECPKPSSLRRQQLIRECPILSNIEPKQIKVWFQNRRCREKQ

>Gh_D13G2409

E-Value: 1.22E-17 Bitscore: 77.1509

KYVRYTAEQVEALERVYAECPKPSSLRRQQLIRECPILSNIEPKQIKVWFQNRRCREKQ

**START domain**

>Gh_A03G0276

E-Value: 1.40E-67 Bitscore: 224.072

PAGLLSIAEETLAEFLSKATGTAVDWVQMPGMKPGPDLVGIFAISQSCSGVAARACGLVSLEPTKIAEILKDRPSWLRDCRNLEVFTMFPAGSGGTIELVYTQMFAPTTLAPARDFWTLRYTTTLENGSFVVCERSLSGSGAGPSTAAAAQFVRAEVLPSGYLIRPCEGGGSIIHIVDHLNLEAWNVPEVLRPLYESSKVIAQKMTIAALRYVKQIA

>Gh_A05G0364

E-Value: 2.17E-66 Bitscore: 219.835

GPDSIGIVAISHGRSGVAARACGLVGLDPTRVAEILKDRPSWYRDCRAVDVINVLSTGNGGTIELLYMQLYAPTTLAPARDFWLLRYTSVMEDGSLVVCERSLNNTQNGPSMPPAANFVRAELLPSGYLIRPCEGGGSIIHIVDHMDLEPWSVPEVLRPLYESSTLLAQKTTMAALRHLRQI

>Gh_A05G0892

E-Value: 8.48E-66 Bitscore: 219.064

PAGLLSIAEETLAEFLSKATGTAVDWVQMPGMKPGPDSVGIFTISQSCSGVAARACGLVSLEPIKIAEILKDRPSWSRDCRNLEVFTMFPAGNGGTIELVYAQKFAPTTLAPARDFWTLRYTTTLENGSLVVCERSLSGSGAGPSAAAAAQFVRAEVLPSGYLIRPCEGGGSIIHIVDHLNLEAWSVPEVLRPLYESSKVIAQKMTIAALRYIRQIA

>Gh_A05G1085

E-Value: 3.03E-78 Bitscore: 252.577

PAGLLSIAEETLAEFLSKATGTAVEWVQMPGMKPGPDSIGIIAISHGCPGVAARACGLVGLEPTRVAELLKDRPSWFHDCRAVDVLNVLPTANGGTIELLYMQLYAPTTLAPARDFWLLRYTSVLEDGSLVVCERSLKNTQNGPSMPPVQHFVRAEMLPSGYLIRPCEGGGSIIHIVDHMDLEPWSVPEVLRPLYESSTVLAQKTTMAALRQLRQIA

>Gh_A06G0833

E-Value: 3.51E-81 Bitscore: 260.281

PAGLLSIAEETLTEFLSKATGTAVEWVQMPGMKPGPDSIGIVAISHGCTGVAARACGLVGLDPTRVAEILKDRPSWYRDCRAVDVINMLSTANGGTIELLYMQLYAPTTLAPARDFWLLRYTSVMEDGSLVVCERSLNNTQNGPSMPPAVNFVRAELLPSGYLIRPCEGGGSIIHIVDHMDLEPWSVPEVLRPLYESSTLLAQKTTMAALRHLRQI

>Gh_A07G0163

E-Value: 6.43E-84 Bitscore: 267.599

PAGLLSIADETLTEFLSKATGTAVEWVQMPGMKPGPDSIGIVAISHGCTGVAARACGLVGLDPTRVAEILKDRPSWFRDCRAVDVINVLSTGNGGTIELLYMQLYAPTTLAPARDFWLLRYTSVLEDGSLVVCERSLNNTQNGPNMPPAANFVRAEMLPSGYLIRPCEGGGSIIHIVDHMDLEPWSVPEVLRPLYESSTLLAQKTTMAALRHLRQI

>Gh_A08G1765

E-Value: 7.11E-70 Bitscore: 230.235

PAGLLSIAEGTLKEFLSKATGTAVDWVQMPGMKPGPDSVGIFAISQSCSGVAARACGLVSLEPTKIAEILKDRPSWFRDCRNLEVFTMFRAGNGGTIELIYTQMFAPTTLAPARDFWTLRYTTTLENGSLVVCERSLSGSGAGPNAAAAAQFVRAEMLTSGYLIRSCEGGGSIVHIVDHLNLEAWSVPEVLRPLYESSKVVAQKMTIAALRYVRQIA

>Gh_A10G0035

E-Value: 9.07E-65 Bitscore: 216.368

PAGLLTIAEETLAEFLSKATGIAVDWVQMIGMKPGPDSIGIVAVSRNCSGVAARACGLVSLEPTKVAEILKDRPSWYRDCRCIDVLSIIPTANGGTIELIYMQTYAPTTLAAARDFWTLRYTTSLEDGSLVICERSLTSSTGGPTGPPTTSFVRAEMLPSGFLIRPCDGGGSIIHIVDHVDLDVWSVPEVLRPLYESSKILAQKMTIAALRHIRQIA

>Gh_A13G2011

E-Value: 3.99E-69 Bitscore: 227.924

PAGLLSIAEETLAEFLSKATGTAVNWVQMPGMKPGPDSVGIFAISQSCSGMAARACGLVSLEPTKIAEILKDRPSWFRDCRKLEVFTMFPAGNGGTIELVYTQMFAPTTLAPARDFWTLRYTTTSENGSLVVCERSLSGSGAGPSVASAAQFVRAEVLPSGYLIRPCEGGGSIIHIVDHLNLEAWSVPEVLRPLYESSRVIAQKMTIPALRYVRQIA

>Gh_D03G1290

E-Value: 1.55E-68 Bitscore: 226.768

PAGLLSIAEETLAEFLSKATGTAVDWVQMPGMKPGPDSVGIIAISQSCSGVAARACGLVSLEPTKIAEILKDRPSWLRDCRNLEVFTMFPAGSGGTIELVYTQMFAPTTLAPARDFWTLRYTTTLENGSFVVCERSLSGSGAGPSTAAAAQFVRAEVLPSGYLIRPCEGGGSIIHIVDHLNLEAWSVPEVLRPLYESSKVIAQKMTIAALRYVKQIA

>Gh_D05G0479

E-Value: 7.16E-82 Bitscore: 262.207

PAGLLSIAEETLTEFLSKATGTAVEWVQMPGMKPGPDSIGIVAISHGRSGVAARACGLVGLDPTRVAEILKDRPSWYRDCRAVDVINVLSTGNGGTIELLYMQLYAPTTLAPARDFWLLRYTSVMEDGSLVVCERSLNNTQNGPSMPPAANFVRAELLPSGYLIRPCEGGGSIIHIVDHMDLEPWSVPEVLRPLYESSTLLAQKTTMAALRHLRQI

>Gh_D05G0975

E-Value: 1.24E-65 Bitscore: 218.679

PAGLLSIAEETLAEFLSKATGTAVDWVQMPGMKPGPDSVGIFTISQSCSGVAARACGLVSLEPVKIAEILKDRPSWSRDCRNLEVFTMFPAGNGGTIELVYAQTFAPTTLAPARDFWTLRYTTTLENGSLVVCERSLSGSGAGPSAAAAAQFVRAEVLPSGYLIRPCEGGGSIIHIVDHLNLEAWSVPEVLRPLYESSKVIAQKMTIAALRYIRQIA

>Gh_D05G1263

E-Value: 4.55E-78 Bitscore: 252.191

PAGLLSIAEETLAEFLSKATGTAVEWVQMPGMKPGPDSIGIIAISHGCPGVAARACGLVGLEPTRVAELLKDRPSWFHDCRAVDVLNVLPTANGGTIELLYMQLYAPTTLAPARDFWLLRYTSVLEDGSLVVCERSLKNTQNGPSMPPVQHFVRAEMLPSGYLIRPCEGGGSIIHIVDHMDLEPWSVPEVLRPLYESSTVLAQKTTMAALRQLRQIA

>Gh_D06G0966

E-Value: 3.51E-81 Bitscore: 260.281

PAGLLSIAEETLTEFLSKATGTAVEWVQMPGMKPGPDSIGIVAISHGCTGVAARACGLVGLDPTRVAEILKDRPSWYRDCRAVDVINMLSTANGGTIELLYMQLYAPTTLAPARDFWLLRYTSVMEDGSLVVCERSLNNTQNGPSMPPAVNFVRAELLPSGYLIRPCEGGGSIIHIVDHMDLEPWSVPEVLRPLYESSTLLAQKTTMAALRHLRQI

>Gh_D07G0220

E-Value: 3.06E-84 Bitscore: 268.37

PAGLLSIADETLTEFLSKATGTAVEWVQMPGMKPGPDSIGIVAISHGCTGVAARACGLVGLDPTRVAEILKDRPSWFRDCRAVDVINVLSTGNGGTIELLYMQLYAPTTLAPARDFWLLRYTSVLEDGSLVVCERSLNNTQNGPNVPPAANFVRAEMLPSGYLIRPCEGGGSIIHVVDHMDLEPWSVPEVLRPLYESSTLLAQKTTMAALRHLRQI

>Gh_D08G2109

E-Value: 1.12E-69 Bitscore: 229.465

PAGLLSIAEGTLKEFLSKATGTAVDWVQMPGMKPGPDSVGIFAISQSCSGVAARACGLVSLEPTKIAEILKDRPSWFRDCRNLEVFTMFRAGNGGTIELIYTQMFAPTTLAPARDFWTLRYTTTLENGSLVVCERSLSGSGAGPNAAAAAQFVRAEMLPSGYLIRSCEGGGSIVHIVDHLNLEAWSVPEVLRPLYESSKVVAQKMTIAALHYIRQIA

>Gh_D10G0038

E-Value: 2.35E-66 Bitscore: 220.605

PAGLLTIAEETLAEFLSKATGTAVDWVQMIGMKPGPDSIGIVAVSRNCSGVAARACGLVSLEPTKVAEILKDRPSWYRDCRCIDVLSIIPTANGGTIELIYMQTYAPTTLAAARDFWTLRYTTSLEDGNLVICERSLTSSTGGPTGPPTTSFVRAEMLPSGFLIRPCDGGGSIIHIVDHVDLDVWSVPEVLRPLYESSKILAQKMTIAALRHIRQIA

>Gh_D13G2409

E-Value: 1.57E-68 Bitscore: 226.383

PAGLLSIAEETLAEFLSKATGTAVNWVQMPGMKPGPDSVGIFAISQSCSGVAARACGLVSLEPTKIAEILKDRPSWFRDCRKLEVFTMFPAGNGGTIELVYTQMFAPTTLAPARDFWTLRYTTTLENGSLVVCERSLSGSGAGPSVASAAQFVRAEVLPSGYLIRPCEGGGSIIHIVDQLNLEAWSVPEVLRPLYESSKVIAQKMTIPALRYVRQIA

**MEKHLA domain**

>Gh_A03G0276

E-Value: 5.12E-63 Bitscore: 208.121

LAQWIYQSYSYHIGAELLRSESLGSDSILKNLWQHQDAILCCSLKSLPVFIFANQAGLDMLETTLVSLQDITLDKIFDEPGRKALCSDFAKLMQQGYAYFPAGICMSTMGRHVSYEQAVAWKVLEADESTVHCLAFSFVNWSF

>Gh_A05G0364

E-Value: 3.36E-67 Bitscore: 218.522

LARWICQSYRCYLGVELLEYEGSESILKTLWHHTDAVLCCSLKALPVFTFANQAGLDMLETTLVALQDISLEKIFDDNGRKALFAEFPQVMQQQGFMCLQGGICLSSMGRPISYERAVAWKVVNDEENAHCICFMFVNWSF

>Gh_A05G0892

E-Value: 1.05E-63 Bitscore: 210.047

LAHWICRSYSYHLGAELLRSESLGGDSILKNLWQHQDAILCCSLKSQPVFIFANQAGLDMLETTLVALQDITLDKLFDESGRKALCSDFGKLMQQGYACLPAGICISTMGRHVSYEQAFAWKVLEADESTVHCLAFSFVNWSF

>Gh_A05G1085

E-Value: 4.40E-69 Bitscore: 224.3

LARWICQSYRVYMGVELLKSGTEGGESVLKTLWHHSDAIMCCSLKALPVFTFANQAGLDMLETTLVALQDLTLEKIFDEHGRKTLCTEFPQIIQQGFACLQGGICLSSMGRPVSYERAVAWKVLNEEENAHCICFMFVNWSF

>Gh_A06G0833

E-Value: 3.68E-65 Bitscore: 213.899

LARWICQSYRCYMGDELLKHEGSESILKVLWHHTDAVLCCSLKALPVFTFANQAGLDMLETTLVSLQDISLEKIFDENGRKTLFTEFPQVMQQGFMCLQGGICLSSMGRPVSYERAVAWKVVNDEENAHCICFTFINWSF

>Gh_A07G0163

E-Value: 3.14E-62 Bitscore: 205.81

LARWICQSYRCFLRMELLKQEGNESILKSLWHHTDAILCCSMKALPIFMFGNQAGLDMLETTLVALQDISLEKIFDENGRKALFAEFPQVMQQQGFMCLQGGICLSSMGRAVSYERAVAWKVVNDEENAHCICFMFINWSF

>Gh_A08G1765

E-Value: 2.55E-60 Bitscore: 200.802

LAHWICRSYSYHFGAELLRPERPVGDSLLKNLWQHQDAILCCSLKPLPVMIFANQAGLDMLETTLMALQDISLDKIFDETGRKTLCSDFAKLMQEGLAYLPAGICMSTMGRHVSYEQAVAWKVLEADESTVHCLAFSFVNWSF

>Gh_A10G0035

E-Value: 3.04E-59 Bitscore: 197.721

LVCWICRSYRIHTGGELLRADSQSGDALLKQLWNHSDAIMCCSLKTNASPVFTFANQAGLDMLETTLVALQDIMLDKILDEAGRKSLCSEFSKIMQQGFAHLPAGICVSSMGRPVSYEQVIVWKVVDDDNDDAANHCLALMFVNWSF

>Gh_A13G2011

E-Value: 1.48E-56 Bitscore: 190.402

LAHWICQSYRQLLKSESLGGDSVLKNLWQHQDAILCCSLKSVPVFIFANQAGLDMLETTLVALQDITLDKIFDELGRKALCFDFTKLMQQGFTHLLAGVCMSTMGRHVSYEQAVAWKVLAADAKTVHCLAFSFINWSF

>Gh_D03G1290

E-Value: 2.56E-60 Bitscore: 200.802

LAQWICQSYRQVASVFVNLRAELLRSESLGSDSILKNLWQHQDAILCCSLKSLPVFIFANQAGLDMLETTLVSLQDITLDKIFDEPGRKALCSDFAKLMQQGYAYFPAGICMSTMGRHVSYEQAVAWKVLEADESTVHCLAFSFVNWSF

>Gh_D05G0479

E-Value: 7.68E-67 Bitscore: 218.522

LARWICQSYRCYLGVELLEYEGSESILKTLWHHTDAVLCCSLKALPVFTFANQAGLDMLETTLVALQDISLEKIFDDNGRKALFAEFPQVMQQQGFMCLQGGICLSSMGRPISYERAVAWKVVNDEENAHCICFMFVNWSF

>Gh_D05G0975

E-Value: 1.24E-65 Bitscore: 218.679

LAHWICRSYSYHLGAELLRSESLGGDSILKNLWQHQDAILCCSLKSQPVFIFANQAGLDMLETTLVALQDITLDKLFDESGRKALCPDFGKLMQQGYACLPAGICMSTMGRHVSYEQAFAWKVLEADESTVHCLAFSFVNWSF

>Gh_D05G1263

E-Value: 1.60E-67 Bitscore: 220.448

LARWICQSYRVYMGVELLKSGTEGGESVLKTLWHHSDAIMCCSLKLFLQALPVFTFANQAGLDMLETTLVALQDLTLEKIFDEHGRKTLCTEFPQIMQQGFACLQGGICLSSMGRPVSYERAVAWKVLNEEENAHCICFMFVNWSF

>Gh_D06G0966

E-Value: 1.22E-65 Bitscore: 215.055

LARWICQSYRCYMGDELLKHEGSESILKVLWHHTDAVLCCSLKALPVFTFANQAGLDMLETTLVSLQDISLEKIFDENGRKTLFAEFPQVMQQGFMCLQGGICLSSMGRPVSYERAVAWKVVNDEENAHCICFTFINWSF

>Gh_D07G0220

E-Value: 4.60E-60 Bitscore: 200.032

LARWICQSYRCFLRMELLKHEGSESILKSLWHHTDAILCCSMKAPPVFMFGNQAGLDMLETTVVALQNVSLEKIFDENGRKALFAEFPQVMQQQGFMCLQGGICLSSMGRAVSYERAVAWKVVNDEENAHCICFMFINWSF

>Gh_D08G2109

E-Value: 7.66E-59 Bitscore: 196.565

LAHWICRSYSYHLGAKLLRPEPAVGDSLLKNLWQHQDAILCCSLKSLPVMIFANQAGLDMLETTLMALQDITLDKIFDETGRKTLCSDFAKLMQEGLAYLPAGICMSTMGRHVSYEQAVAWKVLEADESTVHCLAFSFVNWSF

>Gh_D10G0038

E-Value: 3.57E-58 Bitscore: 195.024

LARWICRSYRIHTGGELLRADSQSGDTLLEQLWNHSDAIMCCSLKTNASPVFTFANQAGLDMLETTLVALQDIMLDKILDEAGRKSLCTEFSKIMQQGFAHLPAGICVSSMGRPLSYEQAIVWKVVDDDNDDAANHCLGLMFVNWSF

>Gh_D13G2409

E-Value: 1.06E-40 Bitscore: 146.104

LAHWICQSYRQFPFGGRVVEIRITWWQLSIEESLATSGCNIVLFVEGTSVPVFIFANQAGLDMLETTLVDLPDITLDKIFDESGRKALCSDFTKLMQQGFTHLLAGVCMSTMGRHVSYEQAVAWKVLAADANTVHCLAFSFINWSF
